# Clinical impact of melatonin on breast cancer patients undergoing chemotherapy; effects on cognition, sleep and depressive symptoms: A randomized, double-blind, placebo-controlled trial

**DOI:** 10.1371/journal.pone.0231379

**Published:** 2020-04-17

**Authors:** Ana Claudia Souza Palmer, Maxciel Zortea, Andressa Souza, Vinicius Santos, Jorge Villanova Biazús, Iraci L. S. Torres, Felipe Fregni, Wolnei Caumo

**Affiliations:** 1 Department of Pharmacology, Universidade Federal do Rio Grande do Sul (UFRGS), Porto Alegre, RS, Brazil; 2 School of Medicine, Universidade Federal do Rio Grande do Sul (UFRGS), Porto Alegre, RS, Brazil; 3 La Salle University Center, Canoas, RS, Brazil; 4 Division of Breast Surgery, Hospital de Clinicas de Porto Alegre (HCPA), Post-graduate Program in Gynecology and Obstetrics, Universidade Federal do Rio Grande do Sul (UFRGS), Porto Alegre, RS, Brazil; 5 Pharmacology Department, Instituto de Ciências Básicas da Saúde, Universidade Federal do Rio Grande do Sul (UFRGS), Porto Alegre, RS, Brazil; 6 Spaulding Neuromodulation Center, Spaulding Rehabilitation Hospital, Harvard Medical School, Boston, MA, United States of America; 7 Pain and Palliative Care Service, Hospital de Clínicas de Porto Alegre (HCPA), Department of Surgery, School of Medicine, Universidade Federal do Rio Grande do Sul (UFRGS), Porto Alegre, RS, Brazil; Betsi Cadwaladr University Health Board, UNITED KINGDOM

## Abstract

This randomized, double-blinded, placebo-controlled trial tested the hypothesis that 20mg of melatonin before and during the first cycle of adjuvant chemotherapy for breast cancer (ACBC) reduced the side effects associated with cognitive impairment. We evaluated the effects of melatonin on cognition, depressive symptoms and sleep quality, and whether these effects were related to serum levels of Brain Derived Neurotrophic Factor (BDNF) and its receptor, tropomyosin kinase B (TrkB). Thirty-six women were randomly assigned to receive melatonin or placebo for 10 days. To evaluate cognitive performance, we used the Trail-Making-Test Parts A and B (A-B), Rey Auditory-Verbal Learning Test (RAVLT), Controlled Oral Word Association Test (COWAT) and an inhibitory task type Go / No-Go. Our results revealed that melatonin improved executive function on TMT scores, enhanced episodic memory (immediate and delayed) and recognition on RAVLT, and increased verbal fluency in the orthographic COWAT. The TMT-A-B(A-B) were negatively correlated with baseline levels of TrkB and BDNF, respectively. At the end of treatment, changes in TrkB and BDNF were inversely associated with depressive symptoms and sleep quality, but not with the TMT scores. These results suggest a neuroprotective effect of melatonin to counteract the adverse effects of ACBC on cognitive function, sleep quality and depressive symptoms.

## Introduction

Cognitive impairment in patients receiving chemotherapy for breast cancer can manifest with acute and/or delayed complications[1,2]. According to Jansen et al.[3], 23% of women with breast cancer had experienced cognitive impairment before chemotherapy. However, this increased to 52% during chemotherapy[3]. The most impacted domains among breast cancer patients are related to visual memory, visuospatial function and verbal learning, with a moderate to large size effect[4–6]. The neurotoxicity associated with chemotherapy for breast cancer is also substantiated by the persistent cognitive deficits related to volume reduction in the hippocampal gray matter one year after treatment completion[7]. Also, there is evidence that the reduction in hippocampal volume is associated with a decrease in cognitive function in patients with major depression[8]. The mechanisms underpinning these symptoms need to be further investigated.

Current evidence points to a central role of inflammatory cascades activated by cancer or chemotherapy on disruptive cognitive and behavioral changes[9]. According to pre-clinical studies, these effects involve an interplay between neuro-inflammation and neuroplasticity states, especially on neurogenesis processes mediated by Brain-Derived Neurotrophic Factor (BDNF)[10]. This neurotrophic factor is essential to long-term potentiation, learning and memory[11], and serves as a critical regulator of synaptic plasticity, neuronal survival and neurogenesis[12]. In fact, BDNF expression in the brain activates many biological functions via the cell surface tropomyosin receptor kinase B (TrkB). Also, the BDNF / TrkB signaling pathway may act as a regulator of carcinogenesis and metastasis[13] and its overexpression may predict a poor clinical outcome and a worse prognosis in patients with breast cancer[14].

Among multiple mechanisms involving neuroplasticity processes and neurotoxicity effects, salient candidates are the pro-inflammatory cytokines. They mediate neuro-inflammatory processes that disrupt the blood-brain barrier with consequent neuronal dysfunction and activation of astrocyte activity, myotoxicity and eventual apoptosis[15]. Increased serum levels of IL-6 and TNF-alfa were found in breast cancer survivors treated with chemotherapy. This finding was correlated with persistent changes in hippocampal structural[16] and reduction in verbal memory processing during chemotherapy infusions[17]. A gap persists in understanding the mechanisms involved in cognitive dysfunction, depressive symptoms and poor sleep quality in breast cancer patients and during the adjuvant chemotherapy for breast cancer (ACBC). In the same way, there is limited evidence regarding neuroprotective treatments to counteract the neurotoxic effects on neuroplasticity processes involving cognitive and emotional dysfunctions.

Melatonin is an hormone secreted by the pineal gland and its main physiological function is to provide information on the photoperiod of the day through mechanisms related to G-protein linked membrane receptors MT1 and MT2[18]. Therefore, it can be considered a physiological sleep regulator, reaching higher plasma levels during the night. According to several studies, exogenous melatonin has demonstrated a positive influence on depressive symptoms and sleep quality in breast cancer patients[19–21]. However, its neuroprotective effect to contra-regulate the neurotoxicity induced by ACBC on cognitive function needs further exploration. Recent investigations point to an impairment of nighttime production of melatonin, which is associated with reduced sleep efficiency, in patients undergoing breast cancer chemotherapy[22]. All together, we hypothesize a central mechanism of ACBC involved in the dysfunction of neural plasticity and that melatonin has neuroprotective qualities. Thus, we evaluated the effect of melatonin prior to the first cycle and during ACBC on cognitive function related to mental flexibility, episodic memory (immediate and delayed), verbal fluency and inhibitory control, and if patient performance is related to the baseline neuroplasticity state assessed by BDNF and TrkB. Also, we evaluated the effect of melatonin on mental flexibility along with the changes on pre- and post-treatment assessed by the delta-value (Δ) of the Trail Making Test (Δ-TMT Part A and B [A-B]; primary outcome), Δ-sleep quality and Δ-depressive symptoms and its relationship with Δ-BDNF and Δ-TrkB.

## Materials and methods

**Fig 1:**
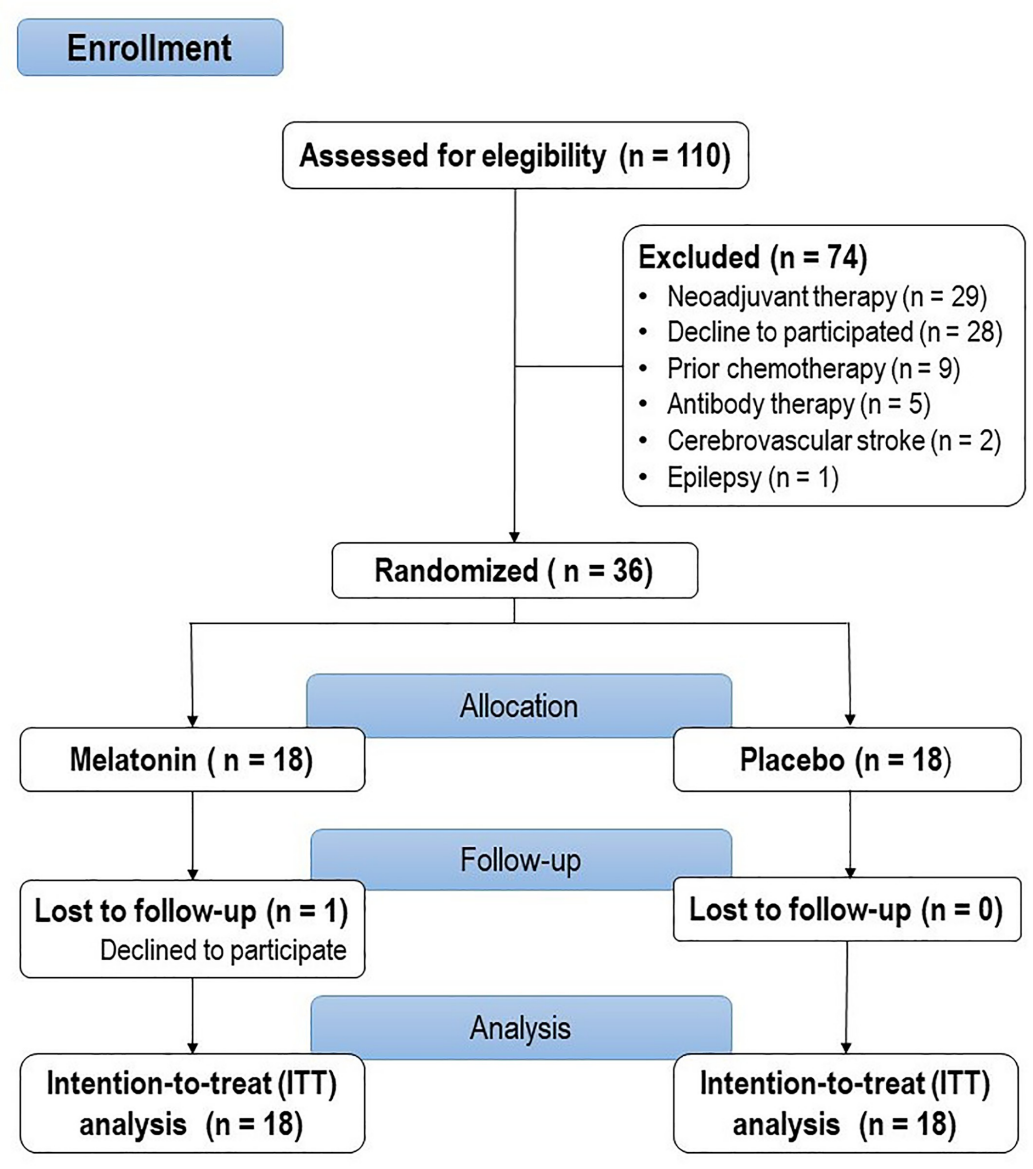
Flowchart of the study.

This trial was structured according to the Consolidated Standards of Reporting Trials (CONSORT) 2010 guideline and was carried out in accordance with the Declaration of Helsinki[23]. The flow of the study is presented in **Fig 1**.

## Study design and eligibility

“This randomized, double-blinded, placebo-controlled trial was approved by the Institutional Review Board of Hospital de Clínicas of Porto Alegre (IRB HCPA/Approval number: 14–0701, May 15, 2015). The study was registered on http://www.clinicaltrials.gov/ (No NCT03205033 Study enrollment start: January 1, 2016 Data collection’s end date: January 1, 2017; delay in registering was related to the person in charge in the group had a time off work). We obtained oral and written informed consent from all patients before participating in this study. The authors confirm that all ongoing and related trials for this drug/intervention are registered.

## Inclusion and exclusion criteria

The patients were selected from the Mastology and Oncology Service at HCPA, which is a public tertiary teaching Medical School located in the South of Brazil. Initially, they were invited to answer a questionnaire to check inclusion and exclusion criteria. All evaluations were performed at the HCPA Clinical Research Center.

We included 36 females, age range 18 to 75 years. *Inclusion criteria*: Patients scheduled for the first cycle of ACBC one month after lumpectomy or mastectomy and with a read and write capacity *Exclusion criteria*: We excluded patients with previous chemotherapy treatment, those who planned for neoadjuvant chemotherapy, and those with another current or prior cancer. We also excluded patients with a history of allergic reaction to melatonin, sleep apnea, diabetes, autoimmune disease (i.e. systemic lupus erythematosus, rheumatoid arthritis, multiple sclerosis, etc.), decompensated liver cirrhosis, severe kidney disease, rotor or Dubin–Johnson syndrome, epilepsy, multiple sclerosis, cerebrovascular stroke, Body Mass Index (BMI) above 35 kg / m^2^, pregnant and breastfeeding.

## Sample size

We estimated the sample size based on previous studies that used the TMT-A-B to assess cognitive flexibility in breast cancer patients[24]. Accordingly, for two dependent variables (TMT-A with a standard deviation equal to 9.64) and (TMT-B with a standard deviation equal to 11.85) with a moderate effect size (f2 = 0.3) to compare melatonin and placebo by MACONVA, with two predictors in a 1:1 ratio, the estimate indicated a sample size of 32 for a power of 90% and an α of 0.01. Considering possible dropouts, we increased the sample by 12.5% so the final sample size comprised of 36 patients (18 per group). The G-Power 3.0.10 software was used to estimate sample size.

## Randomization and masking

Randomization was generated using a computer software. To solve the problem of some researchers or evaluators predicting what the next patients will be assigned for treatment, we used a randomly different block size of 8 and 6. Thirty-six women were allocated to receive melatonin or placebo, an allocation of 1:1. Before the recruitment phase, envelopes containing the protocol materials were prepared by two investigators who made the randomization and they were not involved in the patient’s assessments. Each envelope was sealed and numbered sequentially and included an allocated treatment. The envelope was opened following the sequence of numbers registered in the envelope after the participant consented to participate in the trial. During the entire protocol timeline, the participants, health care providers, research staff, and investigators assessing the outcomes were all blinded to allocation and sequence by receiving numbered sealed envelopes. Further, to assess whether blinding was adequate, at the end of treatment we asked patients to guess the treatment received, with three answer options: melatonin, placebo or unknown.

## Interventions

Patients were randomly assigned to receive melatonin or placebo for ten days, beginning three days prior to the first session of adjuvant chemotherapy. The intervention group received 20 mg of oral melatonin daily approximately 1 hour before bedtime. This dosage has been used previously for neoplasias and other conditions, and showed no important side effects[25]. The placebo group received placebo capsules within the same time. Melatonin capsules were produced using crystalline melatonin with a certificate of purity (M-5250, Sigma Chemical, Saint Louis, MO, USA) from a compounding pharmacy. Placebo capsules contained only cellulose, an indigestible fiber. The tablets and packages were physically identical. The pharmacy packed the melatonin or placebo sequentially numbered in sealed containers. We employed the following strategies to measure adherence to medication use: i) At the end of treatment, a researcher counted the number of tablets consumed during the study period. ii) The patients were asked to record a diary entry if they failed to use the medication. iii) Eligible patients were strongly encouraged to remain on the drug throughout the ten days of treatment, and they visited the clinical center at the end of treatment. Regardless of the patients’ decision to continue or discontinue melatonin after randomization, the patients continued to be assessed during the study period.

## Assessments and instruments

**Fig 2:**
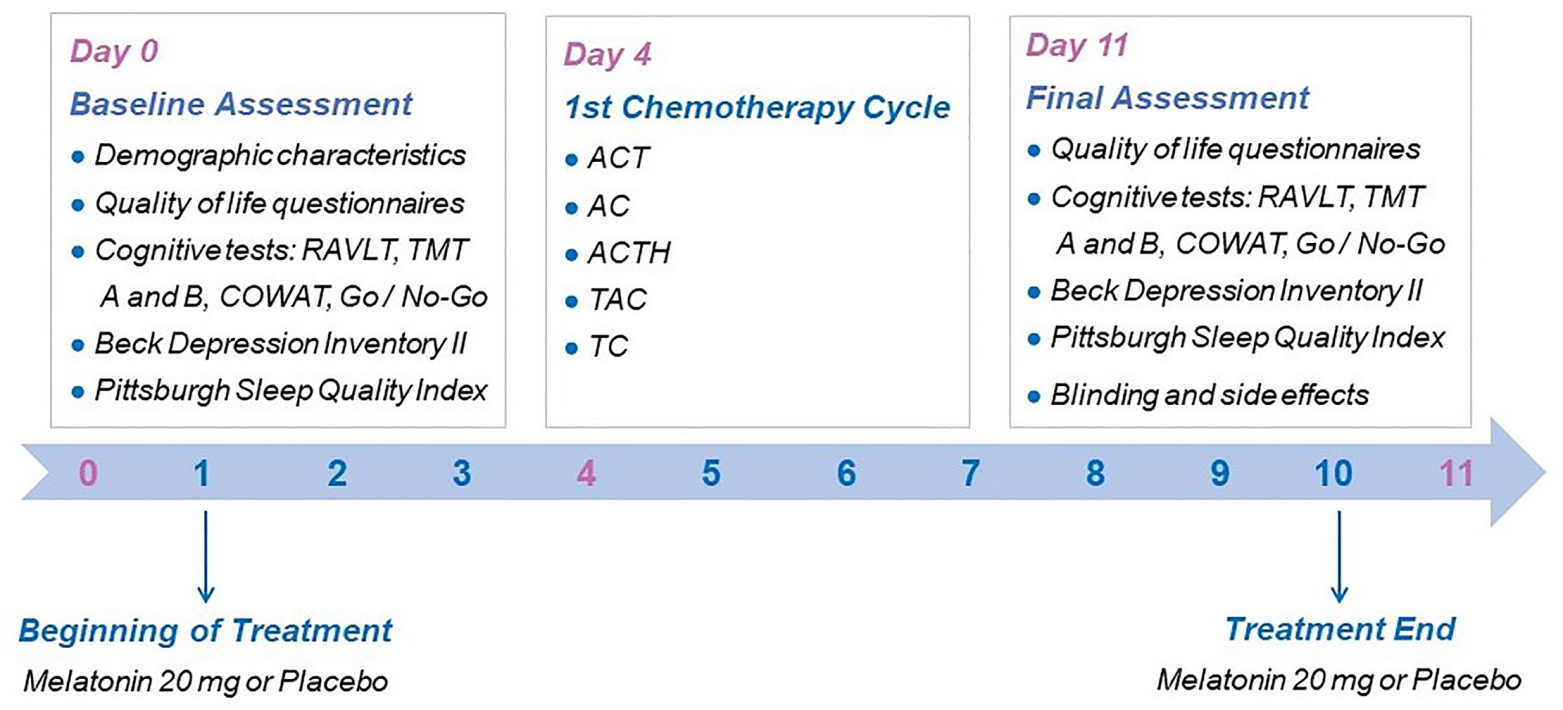
Timeline of assessments and chemotherapy schemes used.

The baseline evaluations were performed up to 4 days prior to the first cycle of chemotherapy. Melatonin was initiated three days prior to the first cycle of adjuvant chemotherapy and continued during and seven days following chemotherapy. Day eight following chemotherapy, the subjects returned for the final evaluation. All assessments were conducted by two independently trained researchers to apply memory and psychological tests. They were monitored by a supervisor psychologist from the Pain and Neuromodulation Research Group at HCPA. The timeline of assessments is presented in **Fig 2**.

## Outcomes

The primary outcome was the total time to accomplish the Trail Making Test Parts A and B (TMT-A-B). Secondary outcomes were the scores from the Rey Auditory-Verbal Learning Test (RAVLT), Controlled Oral Word Association Test (COWAT) and an inhibitory task type Go / No-Go. Additionally, other secondary outcomes were evaluation of depressive symptoms and sleep quality. All tests used to measure cognitive function were selected according to recommendations described by Wefel et al.[26] to harmonize studies in patients with cancer.

### Primary outcomes assessment

#### Trail Making Test (TMT A-B)

The trail-making test is a brief two-part test that evaluates processing speed, divided attention, and cognitive flexibility[27]. The test consists of two parts (A and B). Each part has 25 points on a sheet of paper, which participants connect with a pencil. Part A contains only sequential numbers 1 to 25. Part B consists of numbers and letters alternately mixed: 1 to A, A to 2, 2 to B, and so on. The test results were analyzed as total time to accomplish each part, as well as the proportion and individual differences. Scoring is based on time required to complete the task and number of errors. It has been hypothesized to reflect a wide variety of cognitive processes including attention, visual search and scanning, sequencing and shifting, psychomotor speed, abstraction, flexibility, ability to execute and modify a plan of action, and ability to maintain two trains of thought simultaneously[28].

### Secondary outcomes assessment

#### Cognitive tests

*Rey Auditory-Verbal Learning Test (RAVLT)*. This measures episodic memory, verbal learning, susceptibility to interference (proactive and retroactive), information retention after a certain period of time following performance of other activities, and memory recognition[29]. It is a rapid and direct test, and its use has been widely recognized in the neuropsychological literature. In this study, the adapted test by Malloy-Diniz et al.[30] for the Brazilian population was utilized. In the RAVLT, a list of 15 nouns (list A) is read aloud five consecutive times. Each trial is followed by a spontaneous recovery test. Following the fifth attempt, a list of interferences, which also includes 15 nouns (list B), is read to the patient, followed by recovery (attempt B1). After trial B1, the investigator requested that the patient recall the words from list A without reading it again (attempt A6). To evaluate the learning curve of words during attempts A1 to A5, the learning rate during the attempts is used and are incorporated into the following formula: sum total of A1 to A5. After an interval of 20 to 30 minutes, the patient has to remember the words from list A (tentative A7), without the list being read again. Following the A7 trial, the patient underwent a memory recognition test comprised by reading a list with 15 words from list A, 15 words from list B, and 20 words of distraction (similar to words in list A and B in phonological or semantic terms). With each word read aloud, the patient was asked to indicate whether she belonged to list A or not.

*Controlled Oral Word Association Test (COWAT)*. This test assess lexical knowledge, lexical retrieval ability and executive control abilities[31] and involves word fluency organized into two categories: orthographic and semantic. In orthographic fluency, patients were asked to name as many words as possible, beginning with a certain letter, that is, F, A, and S. Sixty seconds were given for each letter. Patients could not use proper names or words with different tense or suffixes, since the root word was given. In semantic fluency, the patients had to name as many animals as possible in sixty seconds[32].

*Go / No-Go task*. It is a simple and sensitive test of frontal lobe dysfunction, developed to evaluate response inhibition (language and motor function) in a computerized evaluation format. The frequency of Go stimuli relative to No-go is 80%, which maintains a bias and tendency to respond at each trial. The Go / No-Go test was used to measure the capacity for sustained attention and control of responses[33]. On the center of the computer screen were shown a fixation cross (1000 ms) followed by a go letter (e.g., “A”, “G”, “T”, etc.) or a no-go letter (e.g. “H”) for 500 ms. Subjects were instructed to press the “space” key as fast as possible for the go letters and do not press any key for no-go letters (“H”, “X” and “K”). Total task time was 17 minutes. Test instructions were translated to Brazilian Portuguese, but task stimuli and procedures were according to the English version.

#### Clinical measurements: Depressive symptoms and sleep quality

*Beck Depression Inventory (BDI-II)*. Is a questionnaire composed of 21 multiple-choice questions with four options each (0–3). The total BDI score ranges from 0–63, with a higher score indicating a higher degree of depressive symptoms[34]. The BDI-II has generally excellent psychometric properties, including internal consistency coefficient of around 0.9 and retest reliability ranging from 0.73 to 0.96[35] and it has been previously applied in cancer patients[36].

*Pittsburgh Sleep Quality Index (PSQI)*. PSQI is a self-reporting questionnaire that comprises 19-items to assess quality of sleep and identifies sleep disorders. Results are reported with a score ranging from 0 to 21 and is composed of seven domains: (1) subjective quality of sleep; (2) sleep latency, (3) sleep duration; (4) usual sleep efficiency; (5) sleep disorders; (6) use of sleeping pills; and (7) daytime dysfunction. Each item has a response scale ranging from 0 to 3, and lower scores indicate better sleep quality. A total score of 5 or more on the PSQI suggests high sensitivity and specificity of sleep deficiency (Cole et al., 2006). For cancer patients, a cut-off score of 8 was recommended (Carpenter et al., 1998), and a cutoff score of 10 needed to diagnose clinical insomnia[37]. The PQSI reliability and internal validity have been tested in cancer patients[36,38].

#### Biomarkers of neuroplasticity state measured by BDNF and TrkB

*Serum levels of BDNF and TrkB*. Blood samples were collected at the HCPA Clinical Research Center. The samples were centrifuged in plastic tubes for 10 minutes at 4,500 rpm, 4°C and stored at the Unit of Molecular and Protein Analyzes in the HCPA in a −80°C freezer for further BDNF and TrkB assays. Serum-mediator concentrations were determined using BDNF (Chemicon CYT306, lower detection limit 7.8 pg/mL; EMD Millipore, Billerica, MA, USA) and TrkB (MYBI–MBS9346917, lower detection limit 0.25 ng/ml; MyBiosource, San Diego, CA, USA) enzyme-linked immunosorbent-assay kits, according to the manufacturer’s instructions. These serum markers were measured at baseline and after treatment with melatonin.

#### Other instruments and assessments

We used demographic questionnaires to collect data such as age, weight, height, years of study, medications, use of cigarettes, alcohol and other drugs. Also, medical comorbidities were assessed using a standardized questionnaire. To determine side effects related to chemotherapy we applied questionnaires from the European Organization for Cancer Research and Treatment validated for the Brazilian population (EORTC QLQ-C30 and QLQ-BR 23) before and after treatment. EORTC QLQ-C30 assess quality of life in functional scales (physical, role, emotional, cognitive, and social aspects), symptoms scales and items (fatigue, nausea and vomiting, pain, dyspnea, insomnia, appetite loss, constipation, diarrhea, financial difficulties), and a global health score. Low scores on symptoms scales and items and high scores on functional scales and global health indicates better quality of life. The QLQ-BR 23 has four functional scales and a symptom scale and scores are interpreted similarly as EORTC QLQ-C30[39]. The score for each question varies from absent, mild, moderate and severe. The total score converted the sum of these items to a 0 to 100 scale. In breast cancer patients, this instruments revealed an internal consistency Crombach’s alpha of α = 0.82 (global scale) and no difference from test-retest (5 days apart) assessments[39].

## Statistics analysis

Descriptive analysis were performed using mean, standard deviation and frequency. Inferential tests for demographic and clinical measures, as well as for cognitive outcomes, were based on independent sample t-Tests for continuous variables and the Mann-Whitney non-parametric test was used. To control for core cognitive trait of the individual and some imbalance between groups at baseline differences, we assessed changes in cognitive, depression, sleep quality scores and BDNF and TrkB levels based on the mean differences [deltas (Δ-value) that represent the percentual variation of mean [(value at treatment end minus value at baseline)/value at baseline)*100]. To analyze the treatment effect on all primary and secondary outcomes, we conducted multivariate analyses of covariance (MANCOVA). The MANCOVA model was used to examine the influence of BDNF and TrkB levels as modulators of the treatment effectiveness in the Δ-value of the cognitive measurements. The dependent variables were the Δ-value of cognitive tests; the treatment group was the factor, and BDNF and TrkB were covariates. Linear regression analyses to examine the relationship between cognitive flexibility and BDNF and TrkB biomarkers were run when appropriate. A MANCOVA model was also used to examine if the treatment effect on the cognitive flexibility scores, depressive symptoms, and sleep quality was mediated by its effect on neuroplasticity state. The dependent variables of the MANCOVA model were the Δ-Trail Making-Test (TMT-A-B), Δ-BDI-II and Δ-PSQI; the factor was the treatment group, and Δ-BDNF and Δ-TrkB were covariates (see **Table 4**). Bonferroni’s Multiple Comparison adjusted all analyses. We considered all of the randomized patients as part of the analysis using the intention-to-treat (ITT) method, with the worst-case observation carried forward in the respective treatment group (melatonin or placebo). For all analyses, we considered a Type I two-sided error (bicaudal) α<0.05. For statistical analyses, the IBM SPSS Statistics for Windows Version 20.0 was used (IBM Corp., Armonk, NY, USA). The original database with minimal information is available at the S1 File.

## Results

### Socio-demographic and clinical characteristics, blinding and side effects

We selected one hundred and ten women for eligibility and we excluded 74 of them because they did not meet the inclusion criteria. The sample comprised of 36 women scheduled for adjuvant chemotherapy. The characteristics of the participants is presented in **Table 1**. Randomization produced balance groups for most of the characteristics, except in years of school.

**Table 1:** Baseline demographic and clinical characteristics according to treatment group. Data are presented as mean and standard deviation (SD) (n = 36).

*Variables*	*Melatonin (n = 18)*	*Placebo (n = 18)*
Age (years)	54.24 (10.59)	54.11 (9.15)
Formal education (years)	9.29 (4.04)	6.94 (2.57)
Body Mass Index (kg/m^2^)	28.0 (6.14)	29.94 (5.70)
Visual Analogue Scale (0–100)	50 (20.00)	50 (16.48)
Brain-Derived Neurotrophic Factor (ng / mL)	42.92 (17.54)	42.24 (23.95)
Tropomyosin receptor kinase B (ng / mL)	0.48 (0.25)	0.47 (0.50)
Protein S100 Beta (pg / mL)	38.16 (12.42)	32.37 (8.93)
Pittsburgh Sleep Quality Index	8.24 (3.97)	8.44 (2.83)
Beck Depression Inventory II	11.41 (7.73)	10.83 (5.11)
***Chronic disease***		
Hypertension	7 (38.9%) / 11 (61.1%)	8 (44.4%) / 10 (55.6%)
Hypothyroidism	3 (16.7%) / 15 (83.3%)	1 (5.6%) / 17 (94.4%)
Diabetes mellitus	1 (5.6%) / 17 (94.4%)	1 (5.6%) / 17 (94.4%)
Asthma	1 (5.6%) / 17 (94.4%)	1 (5.6%) / 17 (94.4%)
***Psychotropic medication (yes / no)*** *		
Selective serotonin reuptake inhibitors	3 (16.7%) / 15 (83.3%)	3 (16.7%) / 15 (83.3%)
Tricyclics	1 (5.6%) / 17 (94.4%)	2 (11.1%) / 16 (88.9%)
Benzodiazepines	3 (16.7%) / 15 (83.3%)	4 (22.2%) / 14 (77.8%)
Antipsychotics	1 (5.6%) / 17 (94.4%)	________
***Chemotherapy regimens (yes / no)***		
ACT (doxorubicin plus cyclophosphamide followed by weekly paclitaxel) ^1^	9 (50%) / 9 (50%)	9 (50%) / 9 (50%)
AC (doxorubicin plus cyclophosphamide) ^1^	5 (27.8%) / 13 (72.2%)	2 (11.1%) / 16 (88.9%)
ACTH (doxorubicin plus cyclophosphamide followed by paclitaxel plus trastuzumab) ^1^	2 (11.1%) / 16 (88.9%)	3 (16.7%) / (83.3%)
TAC (docetaxel, doxorubicin, and cyclophosphamide) ^2^	1 (5.6%) / 17 (94.4%)	2 (11.1%) / 16 (88.9%)
TC (docetaxel plus cyclophosphamide) ^2^	1 (5.6%) / 17 (94.4%)	2 (11.1%) / 16 (88.9%)

† Mann-Whitney non-parametric test was used. Independent t-tests were applied to all other measures.

* Three patients use more than one psychotropic medication.

Prophylaxis for infusion reactions

^1^ Dexamethasone 20 mg IV 30 minutes before drug administration.

^2^ Dexamethasone 8 mg orally every 12 hours starting one day prior to docetaxel administration.

In the melatonin and placebo group, 13 (54.2%) vs. 11 (45.8%) assumed to have received melatonin, respectively. In the melatonin and placebo group, 4 (44.4%) vs. 5 (55.6%) assumed that they received placebo. Two in the melatonin and 1 in the placebo group assumed to not know their treatment (P = 0.69). Regarding side effects, they were measured by EORTCQLQ-C30 and QLQ-BR 23 and were compared within groups using Wilcoxon Signed Rank test. Melatonin group significantly reduced side effects according to EORTC QLQ-C30 from pre (median [Med] = 30; IQR = ±8) to post-treatment (Med = 23; IQR = ±17)(Z = -3.58; P<0.001) and QLQ-BR 23 (Med = 41; [IQR = ±10] and Med = 36 [IQR = ±13], from pre and post-treatment, respectively) (Z = -2.92; P = 0.004). For the placebo group, there were no significant changes for EORTC QLQ-C30 from pre (Med = 26.5 [IQR = ±12]) to post-treatment (Med = 26.5 [IQR = ±5]) (Z = -1.34; P<0.181) and an increase in symptoms were observed for QLQ-BR 23 (Med = 41.5 [IQR = ±13] and Med = 47 [IQR = ±17] from pre and post-treatment respectively) (Z = -2.68; P = 0.007).

### Univariate analysis of the according to treatment group and their correlations

Mean and standard deviation at baseline and end of treatment of the cognitive tests according to the treatment group as well as their Δ-value are presented in **Table 2**. The t-test for independent samples was used to compare the Δ-value of cognitive measures.

**Table 2:** Cognitive measures at baseline and end of treatment according to melatonin or placebo groups. Data are presented as the mean and standard deviation (SD) (n = 36).

	*Placebo (n = 18)*		*Melatonin (n = 17)*			
	*Mean (SD)*	Δ-value	*Mean (SD)*	Δ-value		P-value*
***Primary outcome***						
Trail Making-Test (TMT-A)						
Baseline	48.06 (29.27)	0.03 (0.29)	44.71 (26.52)	-0.19 (0.13)		0.02
End treatment	49.72 (29.46)		35.14 (18.57)			
Trail Making-Test (TMT-B)						
Baseline	109.50 (55.69)	0.12 (0.19)	122.58 (68.21)	-0.23 (0.16)		<0.001
End treatment	123.72 (60.59)		92.29 (48.25)			
***Secondary outcomes***						
Rey Auditory-Verbal Learning Test *List A1-A5*						
Baseline	42.33 (10.84)	-0.04 (0.27)	39.10 (9.64)	0.25 (0.24)		0.002
End treatment	39.44 (11.99)		47.49 (8.64)			
Rey Auditory-Verbal Learning Test *List A7*						
Baseline	8.33 (4.10)	-0.14 (0.32)	7.82 (3.78)	0.17 (0.22)		0.002
End treatment	6.83 (3.40)		9.62 (4.93)			
Rey Auditory-Verbal Learning Test *Recognition*						
Baseline	42.94 (6.67)	-0.05 (0.10)	44.47 (4.91)	0.06 (0.06)		<0.001
End treatment	40.50 (6.48)		46.89 (3.70)			
Controlled Oral Word Association Test *Orthographic*						
Baseline	30.22 (9.05)	-0.04 (0.27)	32.88 (9.47)	0.24 (0.13)		0.001
End treatment	28.17 (8.62)		39.92 (9.40)			
Controlled Oral Word Association Test *Semantic*						
Baseline	14.44 (3.54)	-0.02 (0.2)	16.89 (4.02)	0.02 (0.21)		0.563
End treatment	14.06 (4.12)		17.05 (4.54)			
Go / No-Go *Hit*						
Baseline	0.84 (0.11)	0.02 (0.14)	0.85 (0.11)	0.03 (0.16)		0.884
End treatment	0.85 (0.12)		0.86 (0.11)			
Go / No-Go *False alarm*						
Baseline	0.16 (0.12)	-0.11 (0.46)	0.16 (0.10)	0.02 (0.77)		0.286
End treatment	0.14 (0.13)		0.15 (0.14)			
Go / No-Go–*D`*						
Baseline	2.27 (0.91)	0.15 (0.41)	2.28 (0.95)	0.17 (0.49)	0.894	
End treatment	2.49 (1.10)		2.46 (1.03)			

Δ-value represent the percentual variation of mean [(value at treatment end minus value at baseline)/value at baseline)*100]

* Correspond to comparisons of Δ-value by the t-test for independent sample.

### Primary and secondary outcomes: Multivariate analysis to compare the treatment group effect on the measurement of cognition according to the neuroplasticity state at baseline assessed by serum TrkB and BDNF

MANCOVA analyses with the Δ-value of cognitive measurements as dependent variables, the factor was the treatment group and the baseline serum level of BDNF and TrkB as covariates are presented in **Table 3**. This analysis revealed significant effects of treatment, Pillai’s Trace’s F (6, 23) = 7.98; p<0.001; η^2^partial = 0.68. Linear regression analysis demonstrated that the TrkB level at baseline was negatively correlated with the Δ-TMT-B-time (Standardized Beta = -0.19; t = -.68, P = 0.001, η^2^partial = 0.32). The serum BDNF level at baseline was negatively correlated with the Δ-TMT-A-time (Standardized Beta = -0.005, t = -2.92 P = 0.007, η^2^partial = 0.24).

**Table 3:** MANCOVA model to compare the treatment effect in the Δ-value of memory measures according to the baseline neuroplasticity state evaluated by the serum BDNF and TrkB (n = 36).

*Corrected Model*	*Type III Sum of Squares*	*df*	*Mean Square*	*F*	*P value*	*η*^*2*^_*partial*_
**Dependent Variables**						
Δ-Trail Making-Test (TMT-A)	0.89^a^	3	0.30	8.07	<0.01	0.47
Δ-Trail Making-Test (TMT-B)	1.19^b^	3	0.40	22.99	<0.01	0.72
Δ-Rey Auditory-Verbal Learning Test *List A1-A5*	0.44^c^	3	0.15	3.2	0.04	0.26
Δ-Rey Auditory-Verbal Learning Test *List A7*	0.89^d^	3	0.30	3.31	0.04	0.27
Δ-Controlled Oral Word Association Test *Semantic*	0.02^e^	3	<0.01	0.17	0.92	0.02
Δ-Controlled Oral Word Association Test *Orthographic*	0.60^f^	3	0.20	3.87	0.02	0.30
***Intercept***						
Δ-Trail Making-Test (TMT-A)	0.13	1	0.13	3.42	0.08	0.11
Δ-Trail Making-Test (TMT-B)	0.04	1	0.04	2.12	0.16	0.07
Δ-Rey Auditory-Verbal Learning Test *List A1-A5*	0.07	1	0.08	1.54	0.23	0.05
Δ-Rey Auditory-Verbal Learning Test *List A7*	0.02	1	0.02	0.19	0.66	<0.01
Δ-Controlled Oral Word Association Test *Orthographic*	<0.01	1	<0.01	0.02	0.88	<0.01
Δ-Controlled Oral Word Association Test *Semantic*	0.11	1	0.11	2.05	0.16	0.07
**Treatment group**						
Δ-Trail Making-Test (TMT-A)	0.54	1	0.54	14.55	<0.01	0.35
Δ-Trail Making-Test (TMT-B)	0.84	1	0.84	48.60	<0.01	0.64
Δ-Rey Auditory-Verbal Learning Test *List A1-A5*	0.43	1	0.43	9.25	<0.01	0.26
Δ-Rey Auditory-Verbal Learning Test *List A7*	0.85	1	0.85	9.45	<0.01	0.26
Δ-Controlled Oral Word Association Test *Orthographic*	<0.01	1	<0.01	0.10	0.75	<0.01
Δ-Controlled Oral Word Association Test *Semantic*	0.56	1	0.56	10.81	<0.01	0.29
***Baseline tropomyosin receptor kinase B (TrkB)***						
Δ-Trail Making-Test (TMT-A)	0.01	1	0.01	0.27	0.60	0.01
Δ-Trail Making-Test (TMT-B)	0.22	1	0.22	12.78	<0.01	0.32
Δ-Rey Auditory-Verbal Learning Test *List A1-A5*	0.04	1	0.04	0.81	0.38	0.03
Δ-Rey Auditory-Verbal Learning Test *List A7*	2.05E	1	2.05E	<0.01	0.99	<0.01
Δ-Controlled Oral Word Association Test *Orthographic*	0.02	1	0.02	0.35	0.56	0.01
Δ-Controlled Oral Word Association Test *Semantic*	0.08	1	0.08	1.52	0.23	0.05
***Baseline Brain Derived Neurotrophic Factor (BDNF)***						
Δ-Trail Making-Test (TMT-A)	0.32	1	0.32	8.55	<0.01	0.24
Δ-Trail Making-Test (TMT-B)	0.01	1	0.01	0.78	0.39	0.03
Δ-Rey Auditory-Verbal Learning Test *List A1-A5*	<0.01	1	<0.01	0.01	0.92	<0.01
Δ-Rey Auditory-Verbal Learning Test *List A7*	0.04	1	0.04	0.44	0.51	0.02
Δ-Controlled Oral Word Association Test *Orthographic*	<0.01	1	<0.01	0.04	0.84	<0.01
Δ-Controlled Oral Word Association Test *Semantic*	<0.01	1	<0.01	0.05	0.83	<0.01

To perform the analysis, we calculated the Δ-value represent the percentual variation [(value at treatment end minus value at baseline)/value at baseline)/value at baseline)*100]

before and after treatment for all outcomes measures.

R Squared = 0.473 (Adjusted R Squared = 0.414) _a_

R Squared = 0.719 (Adjusted R Squared = 0.687) _b_

R Squared = 0.262 (Adjusted R Squared = 0.180) _c_

R Squared = 0.269 (Adjusted R Squared = 0.188) _d_

R Squared = 0.019 (Adjusted R Squared = 0.091) _e_

R Squared = 0.301 (Adjusted R Squared = 0.223) _f_

### Univariate analysis to compare the effect of treatment on the neuroplasticity state assessed by Δ-BDNF, Δ-TrkB and measures of cognitive flexibility (TMT-A-B), depressive symptoms (BDI II) and sleep quality (PSQI)

*Univariate analysis to compare between treatment groups*: *The* Δ*-value of depressive symptoms*, *sleep quality and serum levels of BDNF and TrkB*. The score on BDI-II at baseline and end of treatment and respective Δ-value presented as mean and SD was [11.41(7.73) vs. 6.71(4.57), Δ-value = -4.70 (5.83)] and [10.83 (5.11) vs. 14.56 (7.76), Δ-value = 3.72 (5.21)] (t = -3.62, P<0.001), in the melatonin and placebo group, respectively. The score on the PQSI at baseline and end of treatment and respective Δ-value presented as mean and SD was [8.24 (3.98) vs. 5.06 (3.34), Δ-value = -3.18 (2.00)] and [8.44 (2.83) vs. 11.06 (3.35) (Δ-value = 2.61 (2.06)] (t = -8.40, P<0.001), in melatonin and placebo group, respectively. Serum levels of BDNF at baseline and end of treatment and respective Δ-value presented as mean and SD was [41.65 (17.72) vs. 21.32 (7.190), Δ-value = -0.43 (0.22)] and [40.88 (23.78) vs. 43.76 (17.74), Δ-value = 0.12 (0.20)] (t = -.76, P<0.001), in melatonin and placebo group, respectively. Serum levels of TrkB at baseline and end of treatment and respective Δ-value presented as mean and SD was 0.56 (0.40) vs. 0.41(0.37), Δ-value = -0.19 (0.33) and 0.47 (0.50) vs. 0.52 (0.46), Δ-value = 0.42 (0.65) (t = -.3.42, P = 0.002), in melatonin and placebo group, respectively.

Correlation among the Δ-values of BDNF, TrkB, cognitive flexibility (TMT-A-B), depressive symptoms and sleep quality according to each treatment group and despite the treatment group are presented in Table 4. It is essential to realize that the Δ-values measured the changes in the variables from treatment end minus pre-treatment. Thus, negative Δ-values indicate a decrease in such measures while positive values indicate an increase.

**Table 4:** Correlation between the Δ-values of BDNF, TrkB, cognitive flexibility (TMT-A-B), depressive symptoms and sleep quality (n = 36) according to each treatment group and despite the treatment group.

Placebo group (n = 18)	Δ-BDI-II	Δ-PSQI	Δ- TrkB	Δ-BDNF
Δ-Trail Making-Test (TMT-A)	.538*	-.312	-.234	-.190
Δ-Trail Making-Test (TMT-B)	.269	.121	.221	.138
Δ-Beck Depression Inventory–II (BDI II)		-.235	-.120	-.145
Δ-Pittsburgh Sleep Quality Index (PSQI)			-.041	.098
Δ-Tyrosine Kinase receptor-B (TrkB)				.314
**Melatonin group (n = 18)**				
Δ-Trail Making-Test (TMT-A)	-.397	-.335	.539*	.002
Δ-Trail Making-Test (TMT-B)	.097	.490*	-.340	-.023
Δ-Beck Depression Inventory–II (BDI II)		.318	-.536*	.213
Δ-Pittsburgh Sleep Quality Index (PSQI)			-.670**	-.270
Δ-Tyrosine Kinase receptor-B (TrkB)				.115
**All patients (n = 36)**				
Δ-Trail Making-Test (TMT-A)	.271	.091	.180	.183
Δ-Trail Making-Test (TMT-B)	.504**	.675**	.339*	.549**
Δ-Beck Depression Inventory–II (BDI II)		.538**	.152	.518**
Δ-Pittsburgh Sleep Quality Index (PSQI)			.346*	.655**
Δ-Tyrosine Kinase receptor-B (TrkB)				.553**

*. Correlation is significant at the 0.05 level (2-tailed).

**. Correlation is significant at the 0.01 level (2-tailed).

Δ-value represent the percentual variation of mean [(value at treatment end minus value before treatment)/value at baseline)*100]

### Multivariate analysis to compare Δ-value of cognitive flexibility (TMT-A-B), depressive symptoms and sleep quality considering the effect of treatment on the neuroplasticity biomarkers

The MANCOVA model was used to compare the effect of treatment between groups and its relationship with the neuroplasticity state assessed by Δ-values of BDNF and TrkB. Dependent variables were Δ-value (pre- to post-treatment) on the cognitive flexibility (TMT-A-B), depressive symptoms (BDI II), and the sleep quality (PSQI). The factor was the treatment group, and covariates were Δ-BDNF and Δ-TrkB. To assess if the effect of treatment in the outcomes (TMT-A-B, BDI II and PSQI) was associated with changes in the neuroplasticity state (Δ-BDNF, Δ-TrkB), we analyzed the interaction between groups and Δ-values of the biomarkers of neuroplasticity state. These results are presented in **Table 4**. This analysis revealed significant effects of treatment, Pillai’s Trace’s F (4, 26) = 12.67; p<0.001; η^2^partial = 0.66.

The beta coefficients of MANCOVA analyses with Δ-TrkB as covariate demonstrated that the interaction between the Δ-TrkB and the treatment group was correlated with higher changes in Δ-TrkB compared to placebo, and the Δ-TrkB was correlated with a higher reduction in depressive symptoms (Standardized Beta = -9.31; t = -2.13, P = 0.04, confidence interval (CI 95% = -18.26 to -0.37, η^2^partial = 0.14). In the same way, the interaction between the Δ-TrkB and the treatment group was correlated with higher changes in Δ-TrkB compared to placebo and was also correlated with a higher reduction in the sleep index quality scores (Standardized Beta = -4.50; t = -2.98, P = 0.006, confidence interval (CI 95% = -7.59 to -1.42, η^2^partial = 0.24). However, neither the Δ-BDNF nor its interaction with the treatment group was associated with the effect of treatment on depressive symptoms, sleep and cognitive flexibility assessed by the TMT-A-B.

**Fig 3:**
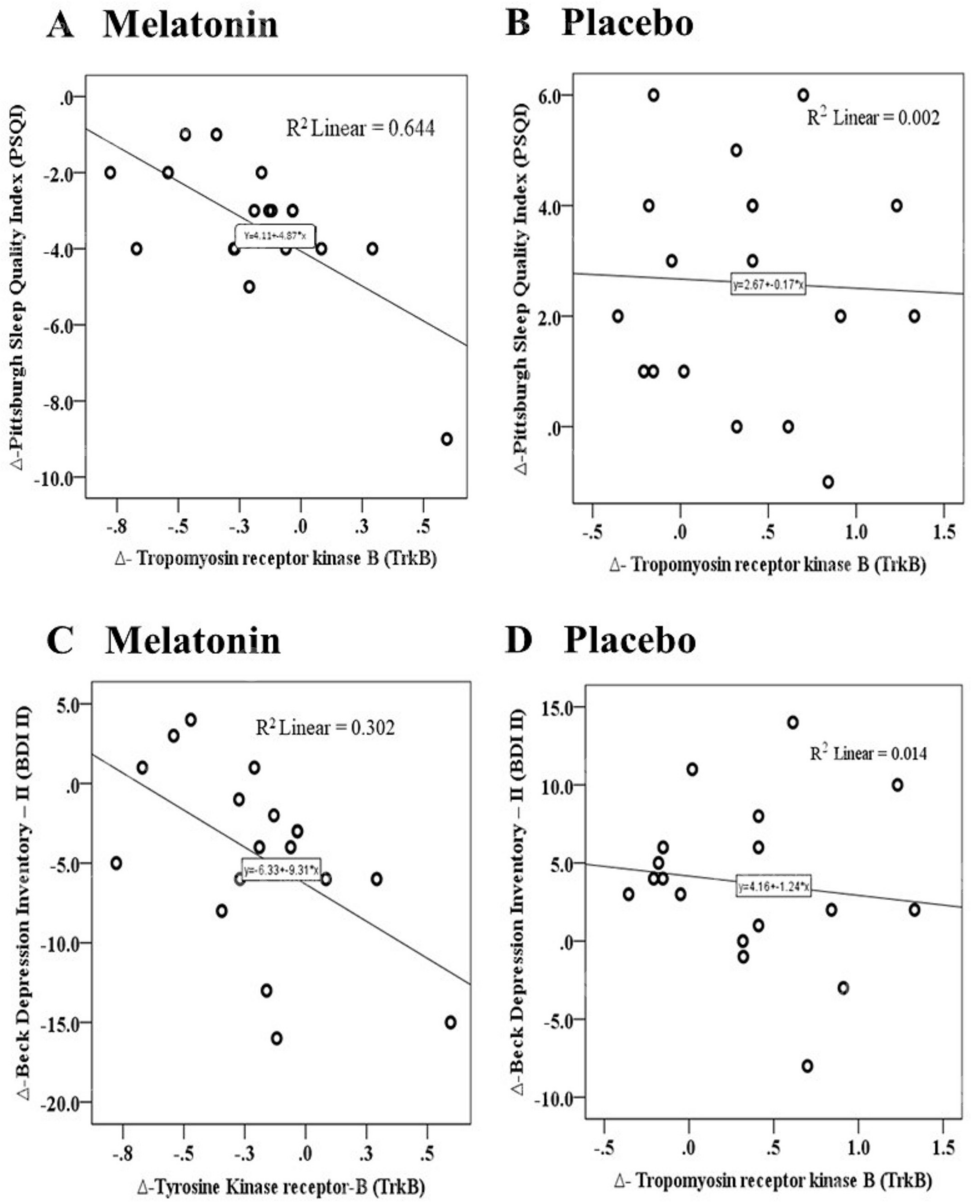
Scatter plots of the Pearson correlation between Δ-TrkB with both Δ-BDI-II (A melatonin, B placebo) and with Δ-PSQI (C melatonin, D placebo).

Scatter plots of the raw Δ-changes on Δ-BDI-II and Δ-PSQI with Δ-TrkB according to placebo and melatonin group is presented in Fig 3A, 3B, 3C and 3D. The Δ-Changes on Δ-TrkB in the melatonin showed a statically significant negative Pearson correlation (r) with Δ-BDI-II and Δ-PSQI. However, in the placebo group we did not observe a correlation with Δ-TrkB, PSQI, nor with BDI-II.

## Discussion

These findings confirm the benefits of melatonin use compared to placebo prior to ACBC in reducing performance time on the TMT-A-B, increasing the score in immediate and delayed recall, and improving recognition in the RAVLT and increasing words recited during the orthographic COWAT. TMT-B and TMT-A were negatively correlated with baseline levels of TrkB and BDNF, respectively. At the end of treatment, TrkB changes were inversely associated with depressive symptoms and sleep quality, but not with TMT-A-B. However, melatonin did not change the capacity for sustained attention and control of responses assessed by the inhibitory control (a Go/No-Go task).

This study extended data that use of 20mg of melatonin prior to the first cycle of ACBC has a neuroprotective effect on cognitive functions evaluated by a set of tests that measures several dimensions of cognitive flexibility allied to attention. However, a study that used 6mg of melatonin prior and post ACBC did not find a similar benefit in cognitive function[19]. In fact, our results are in the sense of most clinical studies that demonstrated a benefit of 20mg of melatonin use in cancer patients on clinical outcomes (i.e. mortality, tumor remission, etc.)[40–42]. Accordingly, two meta-analyses demonstrated benefits of melatonin as an adjuvant to ameliorating radio chemotherapy-related side effects (i.e., asthenia, nausea and vomiting, hypotension, and thrombocytopenia), and improvement of tumor remission and survival[43,44]. Although some of these previous studies reported neuroprotective effects of melatonin, all studies that used melatonin in a dosage of 20mg or higher were not designed to evaluate performance on cognition, depressive symptoms, and sleep quality. In this way, the novelty of this study reveals the benefits of melatonin prior to ACBC on different dimensions of cognition (cognitive flexibility, attention, immediate and delayed episodic memory, executive control and verbal fluency), depressive symptoms and sleep quality allied to the baseline neuroplasticity state and changes in serum BDNF and TrkB induced by melatonin. Indeed, these results showed a statistical difference in these outcome measures, and they have potential clinical relevance to highlight evidence of the neuroprotective effect of melatonin. Additional reasons for the importance of these findings is the scarcity of treatments available to attenuate the side effects of chemotherapy on the central nervous system, as well as the lack of proof of complementary therapies with potential neuroprotective benefit. Thus, melatonin`s properties present an attractive option since it can blunt the most prevalent complaints related to breast cancer chemotherapy, which are comprised of memory deficits, depressive symptoms, and sleep disorders.

Although our results revealed the benefits of melatonin as neuroprotective during ACBC, the underlying mechanism of this is unknown. In fact, this is the first study that investigates the interplay between the neuroplasticity state, neuroprotective effect of melatonin and performance in cognitive flexibility and attention (i.e., TMT-A-B). The trail making test evaluates mainly personal differences in speed and fluid cognitive abilities and both abilities vary according to a particular context[45]. Indeed, in the present study this test was chosen to assert a specific paradigm of executive functioning according to a specific contextual analysis, in this case, the chemotherapy for breast cancer[45]. Although studies generally indicate that large-scale brain networks including prefrontal and parietal structures mediate the trail making test performance, according to Cole and colleagues[46], higher scores on measures of cognitive control (i.e. standard fluid intelligence tests) may be related to a higher degree of global functional connectivity in the lateral prefrontal cortex. Importantly, fluid intelligence tests and the TMT are all based on visual information, for which patients receiving ACBC are found to have an impaired processing[47]. Over this set of the findings related to chemotherapy neurotoxicity, preclinical studies have elicited that melatonin effects can modulate the neuroplastic processes implied in cognitive impairment[48].

Overall, this set of evidence supports our finding that the neuroplasticity state may be considered as a marker to explain the substantial differences between cognitive capacity and impairment in breast cancer patients. Also, it can help to comprehend the susceptibility to neurotoxicity attributed to additive or synergistic mechanisms of anti-cancer drugs. In fact, these results open an avenue to embrace and to investigate variations of cognitive tests in breast cancer patients on chemotherapy, as well, they emphasize the importance to account for the baseline neuroplasticity state as a measure to explore the impact of future melatonin neuroprotective treatment effects. Although our findings suggest a clinical benefit based on clinical measures, the underpinning mechanism is not precise. According to pre-clinical data, the neuroprotective effect of melatonin involves an anti-inflammatory effect[49], while the neurotoxicity of chemotherapy is related to the inflammatory impact[50]. Aligned with this hypothesis exists consistent evidence for a relationship between the influence of pro-inflammatory cytokines and BDNF secretion. Thus, it is possible to hypothesize that the relationship between the baseline BDNF and TrkB with cognitive flexibility may be explained by the influence of pro-inflammatory cytokines on BDNF secretion. However, we cannot affirm that such a specific mechanism explains these clinical benefits. Aligned with this viewpoint, we did not measure peripheral inflammatory mediators (e.g., cytokines). Thus, the interpretation of the relationship between melatonin`s impact on clinical outcomes with its neuroinflammatory effects should be interpreted with parsimony and in a translational perspective based on data of earlier preclinical studies. Accordingly, Dietrich et al.[51] demonstrated that the inflammatory cascade drives many processes of neuroinflammation such as oxidative stress, direct cellular toxicity, and inflammation contributing to altered cellular kinetics in the hippocampus as well as neurovascular blood brain barrier disruption. Even though melatonin´s action is not fully elucidated, previous preclinical studies have reported that melatonin increased activity of natural killer cells (T and B) and cytokine production[52,53]. Also, it demonstrated antiestrogenic effects through the termed MT1 high-affinity G protein-coupled receptors[53–55]. Thus, melatonin´s effect on neuroplasticity processes may be explained partially by its multifaceted anti-inflammatory properties. Besides, it could modulate astrocyte reactivity or death through upregulation of astrocytic anti-oxidative defenses[56]. Overall, we see consistency in existing literature regarding the anti-inflammatory effects of melatonin.

These findings indicate that cognitive flexibility as measured by the TMT-B is correlated with improvement in sleep quality and depressive symptoms (Table 4). Also, they revealed that sleep quality is a determinant to improve the performance when an increased demand for executive function is required as we observed during the TMT-B. Although the explanation for these findings is not clear, they underline the benefits of melatonin to regulate the internal circadian functions. The importance of this effect finds support in a recent study which showed that the first cycle of breast cancer chemotherapy disrupted the sleep-wake activity rhythm, as it decreased both the sleep efficiency and melatonin secretion[22]. Aligned with this perspective, an improvement in sleep quality may be related to a synchronization of the clock genes, since melatonin orchestrates the sleep-wake cycle[57] and according to earlier studies, which found that melatonin's effect entrains the circadian rhythm in patients with metastatic breast cancer[21].

Given our findings, the effects of melatonin on cognitive flexibility are not concurrent with changes in the biomarkers of the neuroplasticity state (**Table 5**). However, the mechanisms involved in the neuroprotective effect are complex and need to be further elucidated. In the same way, the current results need to be interpreted with parsimony because BDNF represents a bridge between inflammation and neuroplasticity. This does not permit us to consider that the effect of melatonin on cognition could be a consequence of its anti-inflammatory properties as there was a previous pre-clinical study in rodents which demonstrated that melatonin elicits all stages of neuroplasticity (i.e., neurogenesis, axogenesis, dendritogenesis, and synaptogenesis)[58]. Indeed, in the present study, the neuroprotective effect of melatonin on neuroplasticity processes is confirmed by a reduction in serum levels of BDNF and TrkB. Furthermore, the relationship between BDNF secretion and the effect of melatonin is further validated in earlier studies of conditions that concur with excessive activation of the stress system associated with inflammation[59] such as in fibromyalgia[60] and endometriosis[61].

**Table 5:** MANCOVA to compare the effect of treatment between groups and its relationship with Δ-values of BDNF and TrkB and cognitive flexibility (TMT-A-B), depressive symptoms and sleep quality (n = 36).

*Corrected Model*	*Type III Sum of Squares*	*df*	*Mean Square*	*F*	*P value*	*η*^*2*^_*partial*_
**Dependent variables**						
Δ-Trail Making-Test (TMT-A)	0.61^a^	5	0.12	2.45	0.06	0.30
Δ-Trail Making-Test (TMT-B)	1.12^b^	5	0.23	7.35	<0.01	0.56
Δ-Pittsburgh Sleep Quality Index (PSQI)	336.3^c^	5	67.26	20.9	<0.01	0.78
Δ-Beck Depression Inventory–II (BDI II)	841.7^d^	5	168.33	6.22	<0.01	0.52
**Intercept**						
Δ-Trail Making-Test (TMT-A)	0.13	1	0.13	2.56	0.12	0.08
Δ-Trail Making-Test (TMT-B)	0.09	1	0.09	3.04	0.09	0.10
Δ-Pittsburgh Sleep Quality Index (PSQI)	9.62	1	9.61	2.99	0.10	0.09
Δ-Beck Depression Inventory–II (BDI II)	2.48	1	2.48	0.09	0.76	<0.01
**Treatment group**						
Δ-Trail Making-Test (TMT-A)	0.03	1	0.03	0.55	0.46	0.02
Δ-Trail Making-Test (TMT-B)	0.24	1	0.24	7.70	0.01	0.21
Δ-Pittsburgh Sleep Quality Index (PSQI)	126.09	1	126.09	39.19	<0.01	0.58
Δ-Beck Depression Inventory–II (BDI II)	157.70	1	157.70	5.83	0.02	0.17
***Δ- Tropomyosin receptor kinase B (TrkB)***						
Δ-Trail Making-Test (TMT-A)	<0.01	1	<0.01	0.015	0.91	<0.01
Δ-Trail Making-Test (TMT-B)	0.03	1	0.03	0.85	0.37	0.03
Δ-Pittsburgh Sleep Quality Index (PSQI)	35.05	1	35.05	10.89	<0.01	0.27
Δ-Beck Depression Inventory–II (BDI II)	169.57	1	169.56	6.27	0.02	0.18
***Δ-Brain derived neurotrophic factor (BDNF)***						
Δ-Trail Making-Test (TMT-A)	0.05	1	0.05	0.99	0.33	0.03
Δ-Trail Making-Test (TMT-B)	<0.01	1	<0.01	0.23	0.63	0.01
Δ-Pittsburgh Sleep Quality Index (PSQI)	<0.01	1	<0.01	<0.01	0.97	<0.01
Δ-Beck Depression Inventory–II (BDI II)	6.03	1	6.03	0.22	0.64	0.01
***Interaction***						
Treatment group * Δ-Tyrosine Kinase receptor-B (TrkB)						
Δ-Trail Making-Test (TMT-A)	0.07	1	0.08	1.48	0.23	0.05
Δ-Trail Making-Test (TMT-B)	<0.01	1	<0.01	0.0	0.91	<0.01
Δ-Pittsburgh Sleep Quality Index (PSQI)	28.63	1	28.63	8.90	0.01	0.24
Δ-Beck Depression Inventory–II (BDI II)	122.7	1	122.69	4.54	0.05	0.14
Treatment group * Δ-Brain derivate neurotrophic factor (BDNF)						
Δ-Trail Making-Test (TMT-A)	0.01	1	0.01	0.23	0.64	0.01
Δ-Trail Making-Test (TMT-B)	0.03	1	0.03	1.07	0.31	0.04
Δ-Pittsburgh Sleep Quality Index (PSQI)	1.66	1	1.66	0.56	0.48	0.02
Δ-Beck Depression Inventory–II (BDI II)	41.43	1	41.43	1.53	0.23	0.05

Δ-value represent the percentual variation of mean [(value at treatment end minus value before treatment)/value at baseline)*100]

R Squared = 0.297 (Adjusted R Squared = 0.175) _a_

R Squared = 0.559 (Adjusted R Squared = 0.483) _b_

R Squared = 0.783 (Adjusted R Squared = 0.745) _c_

R Squared = 0.518 (Adjusted R Squared = 0.434) _d_

Additionally, this study provides evidence that the effect of melatonin on depressive symptoms and sleep quality involves interplay of neuroplasticity processes as demonstrated by changes in Δ-values of BDNF and TrkB. The leading role of melatonin is to regulate sleep and circadian rhythm, and this counteracting circadian misalignment has been proved to be beneficial for the clinical management of mood disorders[62]. It is important to emphasize that in this study, the effects were observed in a sample of women living under intense stressful conditions (i.e. undergoing their first cycle of ACBC), hence, the improvement in these symptoms does not allow definite conclusions regarding melatonin effects as an antidepressant in humans. However, this beneficial effect of melatonin on depressive symptoms and sleep quality are supported by its anti-stress and antidepressant actions. The improvement on sleep quantity produced by melatonin in our study is consistent with previous results found in patients with metastatic breast cancer[21]. This effect is related to melatonin's effect to entrain the circadian rhythm. This is plausible according to a recent study, which found that the first cycle of breast cancer chemotherapy disrupted the sleep-wake activity rhythm and it decreased sleep efficiency and melatonin secretion[22]. The majority of information available about melatonin protective effects involve an interaction with several neurotransmitter systems, including the GABAergic, serotoninergic[63], glutamatergic and nitrergic[64], as well as the modulation of the hypothalamic-pituitary-adrenal axis[65]. As aforementioned, its effect also involve the neuroplasticity mechanism, which can explain the concurrent changes in neuroplasticity markers and the improvement of depressive symptoms and sleep quality in the current study. This hypothesis is supported by preclinical evidence demonstrating that the antidepressant effects of melatonin stimulate dendritogenesis and synaptogenesis in the hippocampus, which is disrupted by chronic psychosocial stress as seen in depression[66]. In the same way, the effects of melatonin on neuroplasticity in the hippocampus attenuated cognitive and memory deficiencies caused by sleep deprivation[67].

We did not observe a significant difference between groups in accuracy or response time on the Go/No-Go task. Several details should be considered in the interpretation of these findings such as the differences observed between groups in regards to task performance and neuropsychological testing, as this does not necessarily suggest that the underlying neurocircuitry is normal. In the same way, these findings could reflect factors related to the nature and difficulty level of the task as well as specific sample characteristics. Consistent with this idea, a review of functional magnetic resonance imaging (fMRI) studies in breast cancer demonstrated a pattern of hyperactivation or recruitment of additional neural resources at low difficulty tasks[68]. However, other studies have shown that when the task difficulty increases, breast cancer patients may be unable to maintain this compensatory response, resulting in decreased neural activation or connectivity[69]. As far as we can discern, the neurobiological process as well as the function of neural networks underpinning the results of these psychometric tests are complex. However, one should realize that the aim of this study was to assess the effects of melatonin on neural networks involved in the inhibitory control mechanism utilizing the psychometric Go/No-Go test. Hence, we cannot associate its pharmacological effects as a response related to a specific biological system, although clinical studies showed that melatonin’s effect involve candidates of the neurobiological systems leading to the response on the Go/No-Go task[70] (i.e., dopaminergic, serotonin, and noradrenaline systems). Overall, our results suggest that the effects of melatonin, according to the protocol of this study, was not sufficient to influence a typical Go/No-Go decision task of sustained attention linked to inhibitory control.

We addressed several limitations concerning our study: First, we agree that our research has an exploratory nature and the results of secondary outcomes should be interpreted as explanatory by increased type I and type II error. Second, although our sample is homogeneous and this is methodologically advantageous, the issue of external validity arises. Third, we assessed serum levels of BDNF and TrKB as measures of the neuroplasticity state, which are less prone to suffer some influences of evaluators. Fourth, a potential limitation is the short-term treatment with melatonin. It would have been difficult to justify a prolonged treatment period in patients undergoing ACBC if they had a high incidence of severe side effects. However, our results are in line with previous studies revealing the benefits of melatonin at improving side effects induced by chemotherapy[55] and with a previous study which found that ten days of melatonin (6 mg), administered 2 hours before bedtime enhanced the ability to recall previously learned information and it improved sleep quality and depressed moods[71]. Considering this rationale, we estimate a long-term administration of melatonin (during 2 or 3 months) would produce similar or larger effect sizes. In association with this short-term treatment, we have to consider that most of the studies linking chemotherapy and cognitive impairment had evaluated these patients after days or months of treatment’s end[72]. More investigation is needed to understand attentional and memory problems during chemotherapy treatment. Fifth, despite the positive evidence of melatonin’s effect on clinical outcomes and neuroplasticity markers, nominally BDNF, S100-B could be related to its anti-inflammatory properties, however it would be speculative to establish a cause-consequence impact. Even though we recognized this limitation, this is a mechanism that finds some support in a previous preclinical study, since a single 150 mg/kg dose intraperitoneally two hours after induction of subarachnoid hemorrhage reduced the levels of interleukins (IL0-1β, IL-6, tumor necrosis factor alpha (TNF-α), vascular endothelial growth factor (VEGF) and matrix metallopeptidases (MMPs) in the left basal cerebral cortex[73]. Also, melatonin administered intraperitoneally at doses of 60 mg/kg, and 50 mg/kg reduced the spinal BDNF concentration[74]. Although these set of results suggest the involvement of anti-inflammatory effects on the clinical outcomes, future studies are required to specifically investigate the relationship between the neuroinflammatory effects and their impact on clinical outcomes. In the same way further studies with more substantial number of patients and a longer treatment duration are needed before defining conclusions regarding melatonin`s impact on patients undergoing ACBC. Finally, all analyses were conducted comparing the Δ-values to adjust for changes within each individual subject, and to promote a better control for personality-related variability in self-reported measures[75].

In conclusion, these findings show the benefit of melatonin use prior and concurrent with the first cycle of ACBC when compared to placebo in improving cognitive flexibility and attention. These cognitive benefits are modulated by the baseline neuroplasticity state as well its neuroprotective effect on depressive symptoms and sleep quality and are allied to changes in neuroplasticity biomarkers assessed by serum BDNF and TrkB. However, since serum cytokine levels fluctuate over time, this observation could be incomplete. It is also possible that MCP-1, which is an early-stage mediator of inflammation, could have led to signaling via other inflammatory pathways that are more indicative of cognitive difficulties at the cycle 2 to cycle 4 time-points.

## Supporting information

S1 Data CONSORT 2010 checklist of information to include when reporting a randomised trial. (DOC)

S2 Data Protocolo De Estudo. (DOCX)

S3 Data Study protocol. (DOCX)

S1 File (XLSX)
