# Supplementary material for: Clinical impact of melatonin on breast cancer patients undergoing chemotherapy; effects on cognition, sleep and depressive symptoms: A randomized, double-blind, placebo-controlled trial
Source: PLoS One. 2020 Apr 17;15(4):e0231379. doi: 10.1371/journal.pone.0231379 (PMC7164654; doi:10.1371/journal.pone.0231379)
Supplement: S2 Data — (DOCX) [file pone.0231379.s002.docx]

**PROTOCOLO DE ESTUDO**

**TÍTULO DO MANUSCRITO:**

**Impacto Clínico da Melatonina em Pacientes com Câncer de Mama Submetidas à Quimioterapia; Efeitos na cognição, sono e sintomas depressivos: Um ensaio clínico, randomizado, duplo-cego, controlado por placebo**

**Informação geral**

• Clinicaltrials.gov número: NCT03205033 (Data de registro: 26 de agosto de 2016)

• Data de recrutamento: janeiro 2016 a janeiro 2017

• Pesquisador Responsável: Wolnei Caumo MD, Ph.D., Professor do Departamento de Dor e Anestesia da Faculdade de Medicina da UFRGS, 2400 Rua Ramiro Barcelos, CEP 90035-003, Porto Alegre, Rio Grande do Sul, Brasil. wcaumo@hcpa.edu.br

• Entidades públicas envolvidas: Laboratório de Dor e Neuromodulação; Serviço de Mastologia e Oncologia. Endereço: Hospital de Clínicas de Porto Alegre, 2350 Rua Ramiro Barcelos, CEP 90035-903, Porto Alegre, Rio Grande do Sul, Brasil.

**Objetivos e hipótese**

• Este ensaio clínico, randomizado, duplo-cego, controlado por placebo testa a hipótese de que 20 mg de melatonina, antes e durante o primeiro ciclo de quimioterapia adjuvante para câncer de mama (ACBC), reduzem a neurotoxicidade associada à quimioterapia.

• Analisar se o efeito do tratamento adjuvante com 20 mg de melatonina é dependente dos níveis séricos de BDNF e TrkB.

• Sugerimos um efeito neuroprotetor da melatonina para contrabalançar os efeitos adversos do ACBC na função cognitiva, qualidade de sono e nos sintomas depressivos.

**Design de estudo**

Randomizado, duplo-cego, paralelo, controlado por placebo com taxa de alocação de 1: 1.

**Metodologia**

Recrutamos 36 pacientes com câncer de mama do serviço de mastologia e oncologia do HCPA. Todas as mulheres com idade entre 18 e 75 anos. Eles foram selecionados de acordo com o regimento de quimioterapia e convidados a participar do estudo de janeiro de 2016 a janeiro de 2017. O tamanho da amostra foi calculado com base em estudos anteriores com dois preditores na proporção de 1: 1, a estimativa indicou um tamanho de amostra de 32 para uma potência de 90% e um α de 0,01. Considerando possíveis desistências, aumentamos a amostra em 12,5% para que o tamanho final da amostra fosse de 36 pacientes (18 por grupo). Os indivíduos foram autorizados a permanecer em medicamentos durante o estudo. Indivíduos com histórico de abuso de substâncias ou evidências de outros distúrbios relacionados à dor foram excluídos.

*Intervenção*: 20 mg de melatonina por dia, aproximadamente 1 hora antes de dormir. O grupo placebo recebeu cápsulas de placebo no mesmo período.

*Randomization.* Antes da fase de recrutamento, a randomização foi gerada usando um sistema de computador por pesquisadores que não administraram a intervenção. Eles colocam a seqüência em envelopes lacrados separadamente opacos. O método de randomização simples foi aplicado, com pacientes designados para um dos dois grupos com uma taxa de 1: 1.

*Cegando.* Os envelopes contendo os números de protocolo dos pacientes foram abertos por um pesquisador auxiliar, a ocultação foi assegurada pela intervenção sendo atribuída somente após a inscrição. Além disso, para avaliar se o mascaramento era eficaz, no final do tratamento foi pedido aos participantes para adivinharem se receberam melatonina ou placebo e para avaliar seu nível de confiança usando uma escala Likert de 5 pontos (de nenhuma confiança a completamente confiante).

*Avaliações* Todos os testes utilizados foram validados para a população brasileira. Para avaliar a função cognitiva, utilizamos o teste Trail Making Parts A e B (TMT-A-B) - Desfecho primário. Desfechos Secundários. Teste de Aprendizagem Auditivo-Verbal de Rey (RAVLT), Teste de Associação de Palavras Controlada (COWAT) e um tipo de tarefa inibitória Go / No-Go. Outros instrumentos utilizados foram: Questionários da Organização Européia de Pesquisa e Tratamento do Câncer validados para a população brasileira (EORTC QLQ-C30 e EORTC QLQ-BR) para avaliar a qualidade de vida e efeitos colaterais, Inventário de Depressão Beck II para avaliar sintomas depressivos, Índice de Qualidade do Sono de Pittsburgh (PSQI) para avaliar a qualidade do sono, para analisar os níveis séricos de BDNF e TrkB foram coletados 10 mL de sangue em um tubo plástico Vacutainer® (BD 366668-1). Os tubos foram centrifugados por 10 minutos a 4500 rpm, 4°C e armazenados em freezer a -80°C. Para as análises utilizamos o Kit de BDNF do Chemicon CYT306, limite inferior de detecção de 7,8 pg / mL; EMD Millipore, Billerica, MA, EUA) e TrkB de MYBI - MBS9346917, limite de detecção inferior 0,25 ng / ml; MyBiosource, San Diego, CA, EUA).

*Teste de trilha (TMT A-B)* O teste consiste em duas partes (A e B, Figura 1). Cada parte tem 25 pontos em uma folha de papel, que os participantes conectam com um lápis. A parte A contém apenas números seqüenciais de 1 a 25. A parte B consiste em números e letras alternadamente misturados: 1 a A, A a 2, 2 a B e assim por diante. Os resultados do teste foram analisados ​​como tempo total para realizar cada parte, bem como a proporção e as diferenças individuais. A pontuação foi baseada no tempo necessário para concluir a tarefa e o número de erros. Diferenças de tempo para completar as duas partes do TMT são geralmente atribuídas a uma diferença nas demandas cognitivas.

*Teste de Aprendizagem Auditivo-Verbal de Rey (RAVLT)* No RAVLT, uma lista de 15 substantivos (lista A) é lida em voz alta cinco vezes consecutivas (Figura 2). Cada ensaio é seguido por um teste de recuperação espontânea. Após a quinta tentativa, uma lista de interferências, que também inclui 15 substantivos (lista B), é lida para o paciente, seguida de recuperação (tentativa B1). Após o julgamento B1, o pesquisador solicitou que o paciente lembre as palavras da lista A sem lê-lo novamente (tentativa A6). Para avaliar a curva de aprendizado de palavras durante as tentativas A1 a A5, é utilizada a taxa de aprendizado durante as tentativas e são incorporadas na seguinte fórmula: soma total de A1 a A5. Após um intervalo de 20 a 30 minutos, o paciente deve lembrar as palavras da lista A (tentativa A7), sem que a lista seja lida novamente. Após o teste A7, o paciente foi submetido a um teste de reconhecimento de memória composto por uma lista com 15 palavras da lista A, 15 palavras da lista B e 20 palavras de distração (semelhantes às palavras da lista A e B em termos fonológicos ou semânticos). Com cada palavra lida em voz alta, o paciente foi solicitado a indicar se ela pertencia à lista A ou não.


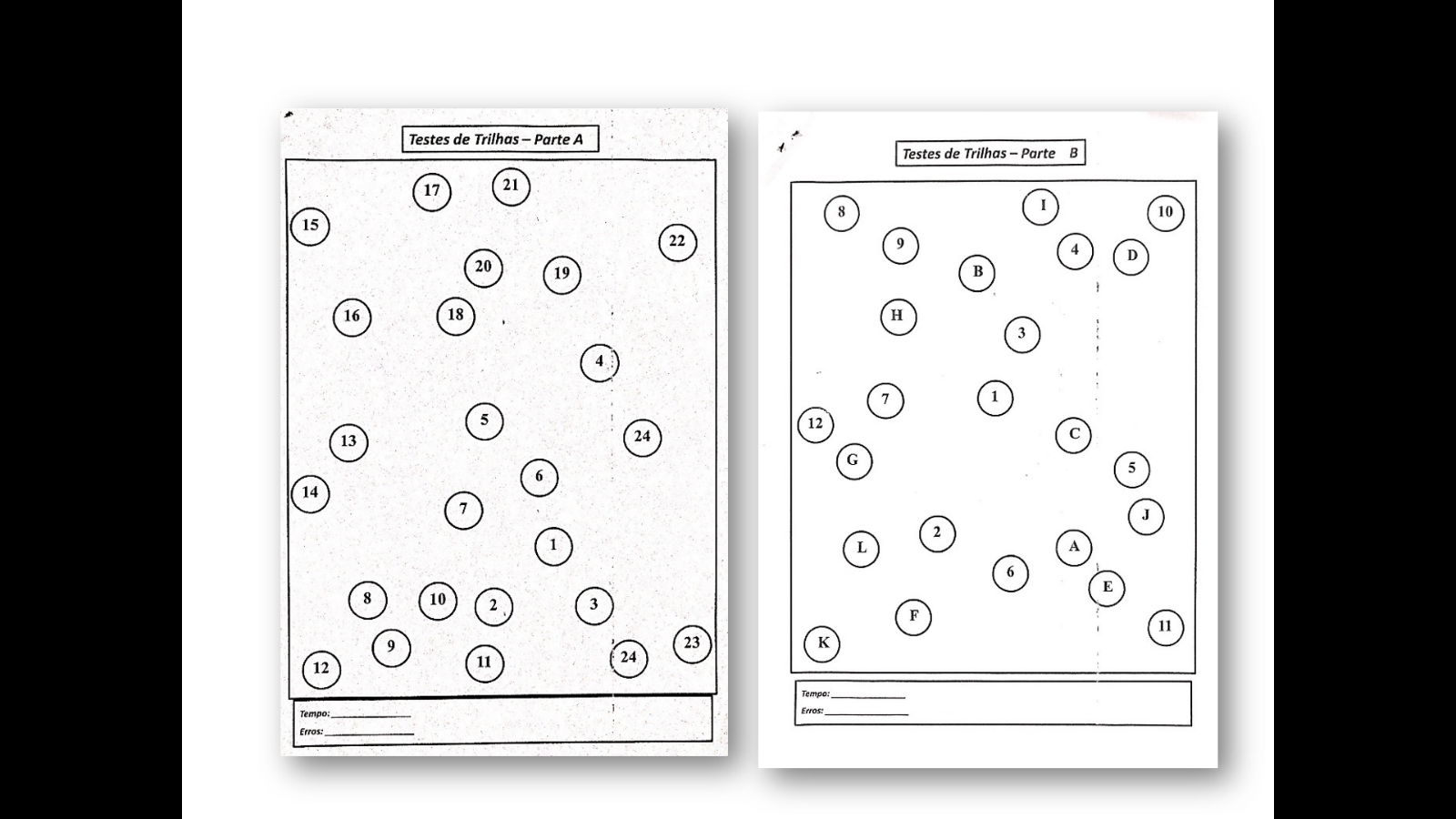


**Figure 1.** Teste de Trilhas Partes A e B


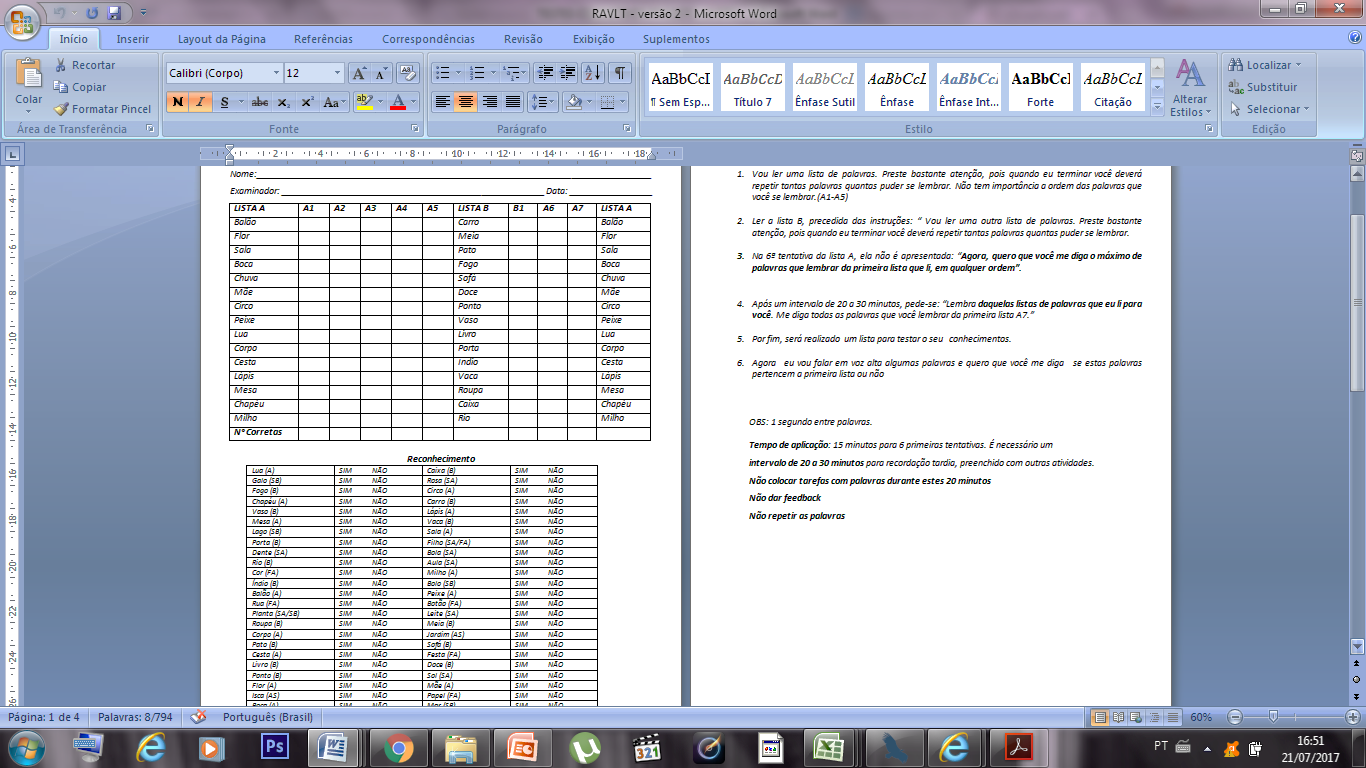


**Figure 2.** Rey Auditory-Verbal Learning Test adaptado para o Português.

*Teste de associação de palavras controladas (COWAT)* Envolve a fluência da palavra organizada em duas categorias: ortográfica e semântica. Na fluência ortográfica, pediu-se aos pacientes que citassem o maior número possível de palavras, começando com uma certa letra, ou seja, F, A e S. Sessenta segundos foram dados para cada letra. Os pacientes não podiam usar nomes próprios ou palavras com diferentes tempos ou sufixos, desde que a raiz da palavra foi dada. Na fluência semântica, os pacientes tiveram que nomear tantos animais quanto possível em sessenta segundos.

*Tarefa Go / No-Go* No centro da tela do computador foi mostrada uma cruz de fixação (1000 ms) seguida por uma letra de go (por exemplo, “A”, “G”, “T”, etc.) ou um não-ir letra (por exemplo, “H”) por 500 ms. Os sujeitos foram instruídos a pressionar a tecla "espaço" o mais rápido possível para as letras e não pressionar qualquer tecla para as letras não-ir ("H", "X" e "K"). O tempo total da tarefa foi de 17 minutos.

**Considerações de segurança**

Aplicamos questionários padronizados (EORTC QLQ-C30 e EORTC QLQ-BR) para avaliar os efeitos adversos da quimioterapia e do tratamento com melatonina. Os sintomas analisados ​​neste estudo foram: boca seca, sensação de mal estar, fogachos, dores de cabeça, fraqueza, falta de apetite, náusea, vômito, constipação, diarréia, cansaço, dificuldade de concentração, preocupação, irritabilidade e dificuldade de memória. A pontuação de cada questão varia de ausente, leve, moderada e grave. Nenhum efeito adverso em relação ao tratamento com melatonina foi relatado durante o julgamento.

**Gerenciamento de dados e análise estatística**

A análise descritiva foi realizada utilizando média, desvio padrão e frequência (%). Testes inferenciais para medidas demográficas e clínicas, bem como para desfechos cognitivos, foram baseados em testes *t* para amostras independentes e para variáveis ​​contínuas o teste não-paramétrico de Mann-Whitney. Para controlar o traço cognitivo central do indivíduo e algum desequilíbrio entre os grupos nas diferenças iniciais, avaliamos a mudança nos escores cognitivos, depressão, qualidade do sono e níveis de BDNF e TrkB com base nas diferenças médias [deltas (Δ-value), média final menos média basal]. Para analisar o efeito do tratamento em todos os desfechos primários e secundários, realizamos análises multivariadas de covariância (MANCOVA). O modelo MANCOVA foi usado para examinar a influência dos níveis de BDNF e TrkB como moduladores da eficácia do tratamento das medidas cognitivas. As variáveis ​​dependentes foram os valores dos testes cognitivos; o grupo de tratamento foi o fator, e BDNF e TrkB foram covariáveis. Análises de regressão linear para examinar a relação entre flexibilidade cognitiva e biomarcadores de BDNF e TrkB foram realizadas quando apropriado. Um modelo MANCOVA também foi usado para examinar se o efeito do tratamento nos escores de flexibilidade cognitiva, sintomas depressivos e qualidade do sono era mediado por seu efeito no estado de neuroplasticidade. As variáveis ​​dependentes do modelo MANCOVA foram: ∆-Trail Making-Test (TMT-A-B), ∆-BDI-II e ∆-PSQI; o fator foi o grupo de tratamento, e ∆-BDNF e ∆-TrkB foram covariáveis ​​(ver Tabela 4). A Comparação Múltipla de Bonferroni ajustou todas as análises. Consideramos todos os pacientes randomizados como parte da análise usando o método de intenção de tratar (ITT), com as piores das observações levadas adiante no respectivo grupo de tratamento (melatonina ou placebo). Para todas as análises, consideramos um erro bilateral de Tipo I (bicaudal) α <0,05. Para análises estatísticas, foi utilizado o IBM SPSS Statistics para Windows Versão 20.0 (IBM Corp., Armonk, NY, U).

**Resultados esperados do estudo**

O tratamento com melatonina pode ter um efeito neuroprotetor na função executiva em comparação ao placebo. Espera-se que o BDNF e o TrkB possam ter um efeito relevante considerando a neuroplasticidade que poderia estar associada ao tratamento com melatonina. Esperamos que o estudo possa apresentar um tratamento adjuvante para neutralizar os efeitos adversos da quimioterapia para o câncer de mama na cognição, sono e depressão.

**Gerenciamento de Projetos**

A.C.S.P. e M.Z. planejou as avaliações, realizou os testes cognitivos e coleta de dados, A.C.S.P .; M.Z. e W.C. projetou e implementou o estudo. M.Z. e W.C. realizou todas as análises estatísticas e interpretação dos resultados. M.Z. monitorou os testes cognitivos. COMO. contribuiu para a randomização. A.C.S.P. e como. fez a preparação da amostra e análise bioquímica. A.C.S.P .; M.Z .; V.S. e W.C. interpretou os resultados e contribuiu para a redação do manuscrito. J.V.B. ajudou a avaliar pacientes do serviço de oncologia e mastologia. I.L.S.T. e F.F. forneceu feedback crítico e ajudou a moldar a pesquisa e o manuscrito.

**Ética e formulários de consentimento informado**

Todos os indivíduos forneceram consentimento informado por escrito antes de participar. O estudo foi aprovado pelo Comitê de Ética em Pesquisa do Hospital de Clínicas de Porto Alegre (HCPA) (Institutional Review Board IRB 14-0701). Segue anexo.
