# Supplementary material for: Clinical impact of melatonin on breast cancer patients undergoing chemotherapy; effects on cognition, sleep and depressive symptoms: A randomized, double-blind, placebo-controlled trial
Source: PLoS One. 2020 Apr 17;15(4):e0231379. doi: 10.1371/journal.pone.0231379 (PMC7164654; doi:10.1371/journal.pone.0231379)
Supplement: S3 Data — (DOCX) [file pone.0231379.s003.docx]

**STUDY PROTOCOL**

**MANUSCRIPT TITLE:**

Clinical Impact of Melatonin on Breast Cancer Patients Undergoing Chemotherapy; Effects on Cognition, Sleep and Depressive Symptoms: A Randomized, Double-Blind, Placebo, Controlled Trial

**General information**

- Clinical Impact of Melatonin on Breast Cancer Patients Undergoing Chemotherapy; Effects on Cognition, Sleep and Depressive Symptoms: A Randomized, Double-Blind, Placebo, Controlled Trial
- Clinicaltrials.gov number: NCT03205033 (Date of registration: August 26, 2016)
- Date of recruitment: January 2016 to January 2017
- Responsible researcher: Wolnei Caumo MD, Ph.D., Professor of Pain and Anesthesia in Surgery Department, School of Medicine at UFRGS, 2400 Ramiro Barcelos Street, Zip code 90035-003, Porto Alegre, Rio Grande do Sul, Brazil. wcaumo@hcpa.edu.br
- Public entities involved: Laboratory of Pain & Neuromodulation; Mastology and Oncology Service. Address: Hospital de Clínicas de Porto Alegre, 2350 Ramiro Barcelos Street, Zip Code 90035-903, Porto Alegre, Rio Grande do Sul, Brazil.

**Aims and hypothesis**

- This randomized, double-blinded, placebo-controlled trial test the hypothesis that 20 mg of melatonin before and during the first cycle of adjuvant chemotherapy for breast cancer (ACBC) reduce the neurotoxicity associate with the chemotherapy.
- Analyze if the effect of adjuvant treatment with melatonin 20 mg is dependent on the serum levels of BDNF and TrkB.
- We suggest a neuroprotective effect of melatonin to counteract the adverse effects of ACBC on cognitive function, sleep quantity and depressive symptoms.

**Study design**

Randomized, double-blind, placebo-controlled, two arm parallel clinical trial design with allocation ratio of 1:1. The flowchart below shows the design scheme.


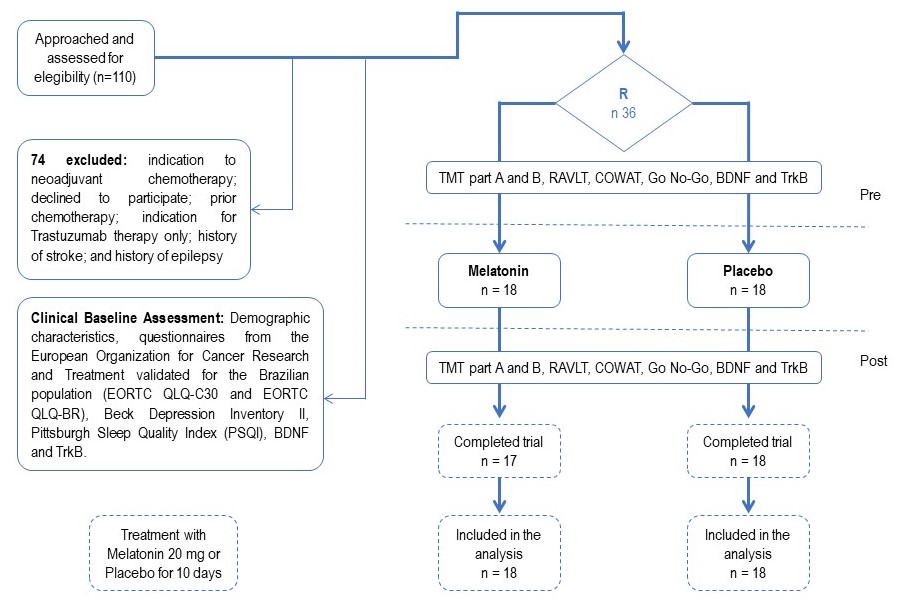


**Methodology**

We recruited 36 breast cancer patients from the mastology and oncology service of the HCPA. All women aged between 18 and 75 years-old. They were select according to the chemotherapy regiment and invited to participate the study from January 2016 to January 2017. Sample size was calculated based on previous studies with two predictors in a 1:1 ratio, the estimate indicated a sample size of 32 for a power of 90% and an α of 0.01. Considering possible dropouts, we increased the sample by 12.5% so the final sample size comprised of 36 patients (18 per group). Subjects were allowed to remain on medications during the study. Subjects with history of substance abuse or evidence of other pain-related disorder were excluded.

Intervention: 20 mg of oral melatonin daily approximately 1 hour before bedtime. The placebo group received placebo capsules within the same time.

*Randomization.* Before the recruitment phase, the randomization was generated using a computer system by researchers who did not administer the intervention. They put the sequence in separately opaque sealed envelopes. The simple randomization method was applied, with patients assigned to the one of the two groups with a rate of 1:1.

*Blinding.* Envelopes containing the patients’ protocol numbers were opened by an auxiliary researcher, concealment was assured by intervention being assigned only after enrollment. Furthermore, to assess whether blinding was effective, at the end of the treatment subjects were asked to guess whether they had received melatonin or placebo and to rate their confidence level using a 5-point Likert scale (from no confidence at all to completely confident).

*Assessments* All tests used have been validated for the Brazilian population. To evaluate cognitive function, we used the Trail Making Test parts A and B (TMT-A-B) – *Primary outcome.*

*Secundary outcomes.* Rey Auditory-Verbal Learning Test (RAVLT), Controlled Oral Word Association Test (COWAT) and an inhibitory task type Go / No-Go. Other instruments used were: Questionnaires from the European Organization for Cancer Research and Treatment validated for the Brazilian population (EORTC QLQ-C30 and EORTC QLQ-BR) to assess quality of life and side effects, Beck Depression Inventory II to assess depressive symptoms, Pittsburgh Sleep Quality Index (PSQI) to asses sleep quality, to analyze the serum levels of BDNF and TrkB we collected 10 mL of blood in a plastic Vacutainer® tube (BD 366668-1). The tubes were centrifuged for 10 minutes at 4,500 rpm, 4°C and stored in −80°C freezer for further assays. For the analyses we used BDNF from Chemicon CYT306, lower detection limit 7.8 pg/mL; EMD Millipore, Billerica, MA, USA) and TrkB from MYBI – MBS9346917, lower detection limit 0.25 ng/ml; MyBiosource, San Diego, CA, USA).

*Trail Making Test (TMT A-B)* The test consists of two parts (A and B, **Figure 1**). Each part has 25 points on a sheet of paper, which participants connect with a pencil. Part A contains only sequential numbers 1 to 25. Part B consists of numbers and letters alternately mixed: 1 to A, A to 2, 2 to B, and so on. The test results were analyzed as total time to accomplish each part, as well as the proportion and individual differences. Scoring is based on time required to complete the task and number of errors. Time differences to complete the two parts of the TMT are usually attributed to a difference in cognitive demands.

*Rey Auditory-Verbal Learning Test (RAVLT)* In the RAVLT, a list of 15 nouns (list A) is read aloud five consecutive times (**Figure 2**). Each trial is followed by a spontaneous recovery test. Following the fifth attempt, a list of interferences, which also includes 15 nouns (list B), is read to the patient, followed by recovery (attempt B1). After trial B1, the investigator requested that the patient recall the words from list A without reading it again (attempt A6). To evaluate the learning curve of words during attempts A1 to A5, the learning rate during the attempts is used and are incorporated into the following formula: sum total of A1 to A5. After an interval of 20 to 30 minutes, the patient has to remember the words from list A (tentative A7), without the list being read again. Following the A7 trial, the patient underwent a memory recognition test comprised by reading a list with 15 words from list A, 15 words from list B, and 20 words of distraction (similar to words in list A and B in phonological or semantic terms). With each word read aloud, the patient was asked to indicate whether she belonged to list A or not.


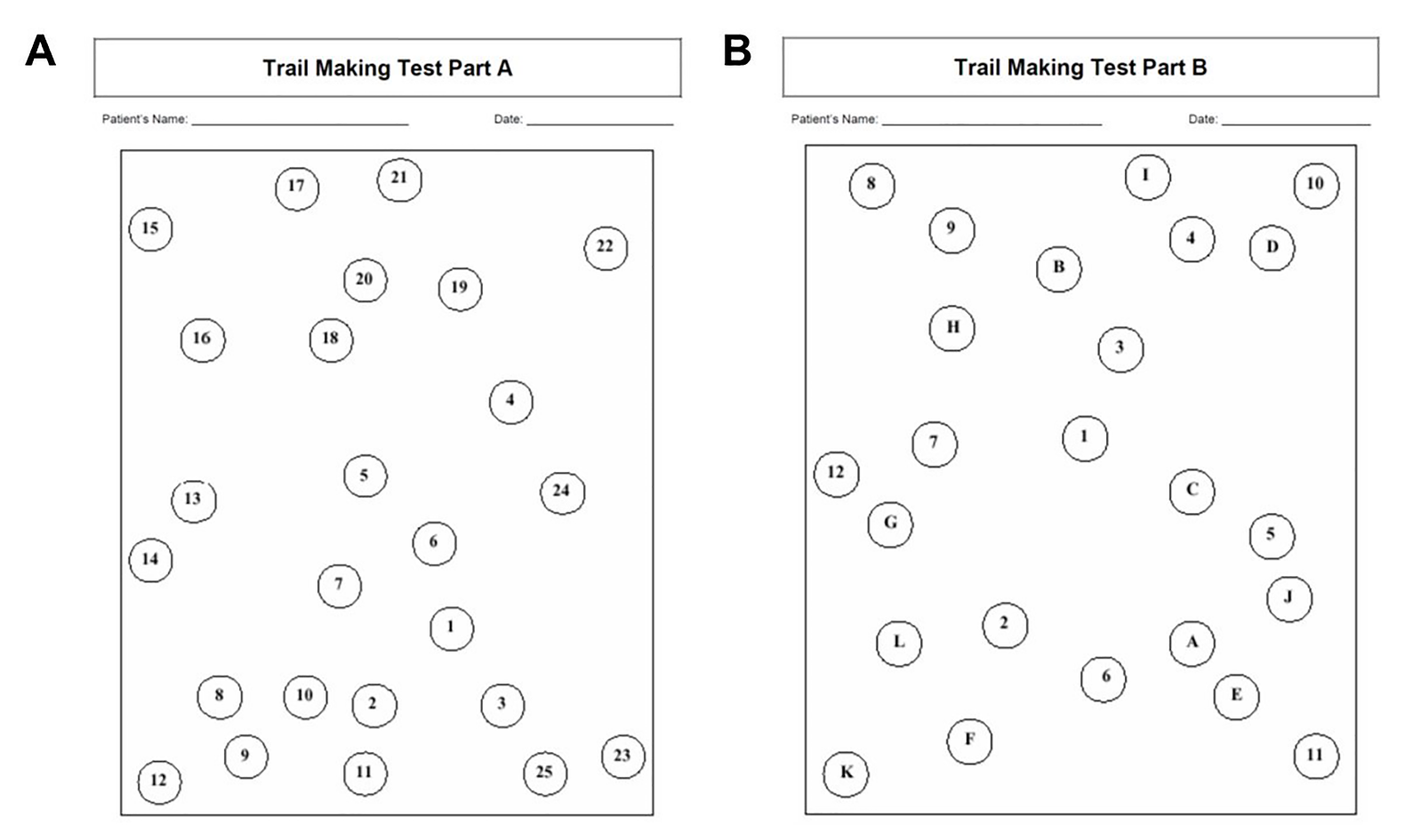


**Figure 1.** Trial Making test parts A and B.


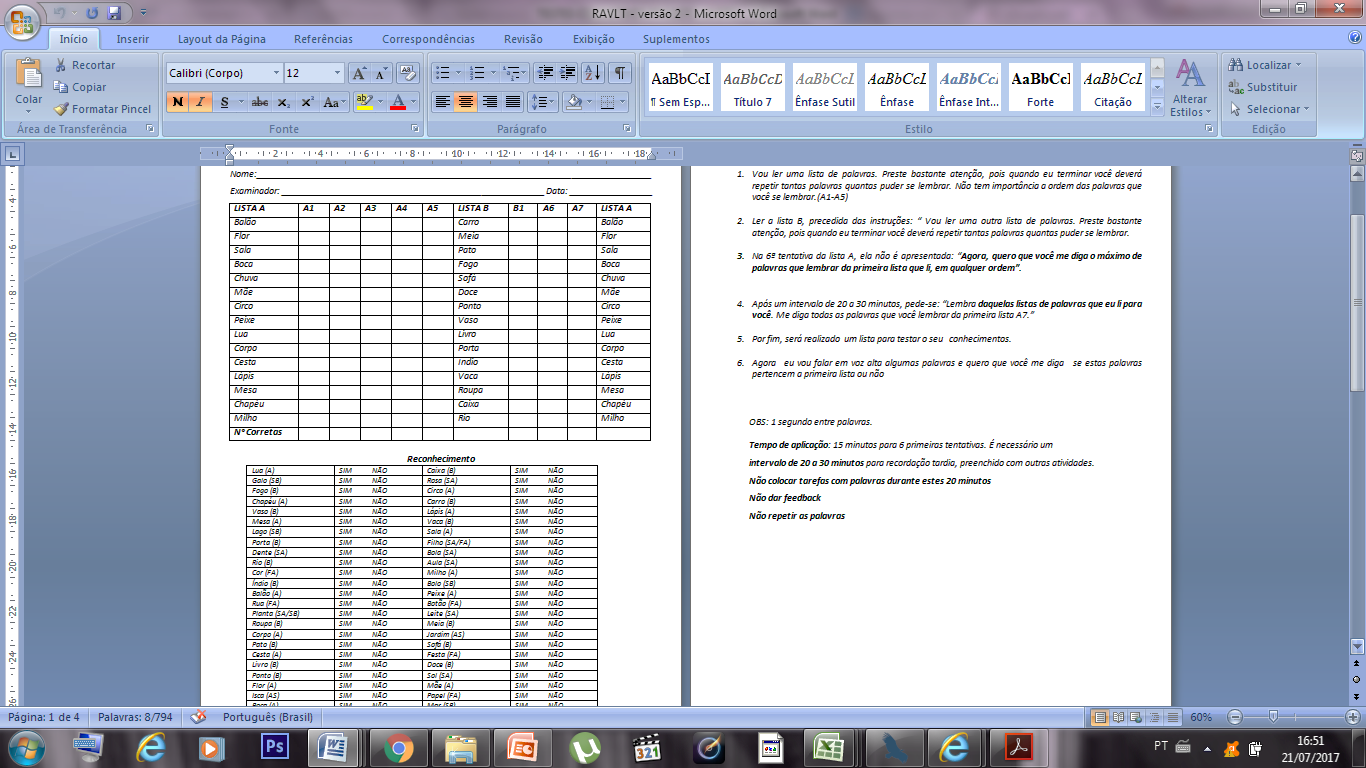


**Figure 2.** Rey Auditory-Verbal Learning Test in adapted to Portuguese.

*Controlled Oral Word Association Test (COWAT) I*nvolves word fluency organized into two categories: orthographic and semantic. In orthographic fluency, patients were asked to name as many words as possible, beginning with a certain letter, that is, F, A, and S. Sixty seconds were given for each letter. Patients could not use proper names or words with different tense or suffixes, since the root word was given. In semantic fluency, the patients had to name as many animals as possible in sixty seconds.

*Go / No-Go Task* On the center of the computer screen were shown a fixation cross (1000 ms) followed by a go letter (e.g., “A”, “G”, “T”, etc.) or a no-go letter (e.g. “H”) for 500 ms. Subjects were instructed to press the “space” key as fast as possible for the go letters and do not press any key for no-go letters (“H”, “X” and “K”). Total task time was 17 minutes.

**Safety Considerations**

We have applied a standardized questionnaires (EORTC QLQ-C30 and EORTC QLQ-BR) to evaluate adverse effects due to chemotherapy and the treatment with melatonin. The symptoms analyzed in this study composed of dry mouth, sick feeling, hot flushes, headaches, weakness, lack of appetite, nausea, vomit, constipation, diarrhea, tiredness, difficulty concentrating, worriedness, irritability and memory difficulty. The score for each question varies from absent, mild, moderate and severe. No adverse effect regarding melatonin treatment was reported during the trial.

**Data Management and Statistical Analysis**

Descriptive analysis were performed using mean, standard deviation and frequency (%). Inferential tests for demographic and clinical measures, as well as for cognitive outcomes, were based on independent sample t-Tests for continuous variables and the Mann-Whitney non-parametric test was used. To control for core cognitive trait of the individual and some imbalance between groups at baseline differences, we assessed change in cognitive, depression, sleep quality scores and BDNF and TrkB levels based on the mean differences [deltas (Δ-value), mean at treatment end minus mean prior treatment]. To analyze the treatment effect on all primary and secondary outcomes, we conducted multivariate analyses of covariance (MANCOVA). The MANCOVA model was used to examine the influence of BDNF and TrkB levels as modulators of the treatment’s effectiveness in the ∆-value of the cognitive measurements. The dependent variables were the ∆-value of cognitive tests; the treatment group was the factor, and BDNF and TrkB were covariates. Linear regression analyses to examine the relationship between cognitive flexibility and BDNF and TrkB biomarkers were run when appropriate. A MANCOVA model was also used to examine if the treatment effect on the cognitive flexibility scores, depressive symptoms, and sleep quality was mediated by its effect on neuroplasticity state. The dependent variables of the MANCOVA model were the ∆-Trail Making-Test (TMT-A-B), ∆-BDI-II and ∆-PSQI; the factor was the treatment group, and ∆-BDNF and ∆-TrkB were covariates (see Table 4). Bonferroni’s Multiple Comparison adjusted all analyses. We considered all of the randomized patients as part of the analysis using the intention-to-treat (ITT) method, with the worst-case observation carried forward in the respective treatment group (melatonin or placebo). For all analyses, we considered a Type I two-sided error (bicaudal) α< 0.05. For statistical analyses, the IBM SPSS Statistics for Windows Version 20.0 was used (IBM Corp., Armonk, NY, U).

**Expected Outcomes of the Study**

The treatment with melatonin may have a neuroprotective effect on executive function compared to placebo. It is expected that BDNF and TrkB may have a relevant effect considering the neuroplasticity that could be associated with the melatonin treatment. We expect the study can present an adjuvant treatment to counteract the adverse effects of chemotherapy for breast cancer on cognition, sleep and depression.

**Project Management**

A.C.S.P. and M.Z. planned the assessments, carried out the cognitive tests and data collection, A.C.S.P.; M.Z. and W.C. designed and implemented the study. M.Z. and W.C. conducted all statistical analyses and interpretation of the results. M.Z. monitored the cognitive tests. A.S. contributed to randomization. A.C.S.P. and A.S. did the sample preparation and biochemical analysis. A.C.S.P.; M.Z.; V.S. and W.C. interpreted the results and contributed to the writing of the manuscript. J.V.B. helped assessing patients from the oncology and mastology service. I.L.S.T. and F.F. provided critical feedback and helped shape the research and the manuscript. W.C. conceived of the presented idea, was involved in planning and supervising the study.

**Ethics and informed consent forms**

All subjects provided written informed consent before participating. The study was approved by the Research Ethics Committee at Hospital de Clínicas de Porto Alegre (HCPA) (Institutional Review Board IRB 14-0701). Attached in the submission archives is the written informed consent.

**Budget**

Multiple institutions contributed to the study funding material. Grants and material came from the following Brazilian agencies: (i) Committee for the Development of Higher Education Personnel (A129/2013 and CAPES PNPD [M.Z. Grant to 2015)]. National Council for Scientific and Technological Development [(CNPq (Grant to I.L.S.T. 302345/2011-6 and W.C. 301256/2013-6)]. Post graduate Research Group at the Hospital de Clínicas de Porto Alegre (HCPA) number: 14-0701. Foundation for Support of Research at Rio Grande do Sul (FAPERGS). Brazilian Innovation Agency (FINEP) process number - 1245/13 (I.L.S.T. and W.C.).
